# Insulin Sensitivity in Adipose and Skeletal Muscle Tissue of Dairy Cows in Response to Dietary Energy Level and 2,4-Thiazolidinedione (TZD)

**DOI:** 10.1371/journal.pone.0142633

**Published:** 2015-11-16

**Authors:** Afshin Hosseini, Muhammad Rizwan Tariq, Fernanda Trindade da Rosa, Julia Kesser, Zeeshan Iqbal, Ofelia Mora, Helga Sauerwein, James K. Drackley, Erminio Trevisi, Juan J. Loor

**Affiliations:** 1 Department of Animal Sciences and Division of Nutritional Sciences, University of Illinois, Urbana, Illinois 61801, United States of America; 2 University of Bonn, Bonn, NRW 53115, Germany; 3 National Autonomous University of Mexico, Querétaro, Querétaro 76230, Mexico; 4 Istituto di Zootecnica, Facoltà di Scienze Agrarie Alimentari ed Ambientali, Università Cattolica del Sacro Cuore, Piacenza 29122, Italy; Max-Delbrück Center for Molecular Medicine (MDC), GERMANY

## Abstract

The effects of dietary energy level and 2,4-thiazolidinedione (TZD) injection on feed intake, body fatness, blood biomarkers and TZD concentrations, genes related to insulin sensitivity in adipose tissue (AT) and skeletal muscle, and peroxisome proliferator-activated receptor gamma (PPARG) protein in subcutaneous AT (SAT) were evaluated in Holstein cows. Fourteen nonpregnant nonlactating cows were fed a control low-energy (CON, 1.30 Mcal/kg) diet to meet 100% of estimated nutrient requirements for 3 weeks, after which half of the cows were assigned to a higher-energy diet (OVE, 1.60 Mcal/kg) and half of the cows continued on CON for 6 weeks. All cows received an intravenous injection of TZD starting 2 weeks after initiation of dietary treatments and for an additional 2 weeks, which served as the washout period. Cows fed OVE had greater energy intake and body mass than CON, and TZD had no effect during the administration period. The OVE cows had greater TZD clearance rate than CON cows. The lower concentration of nonesterified fatty acids (NEFA) and greater concentration of insulin in blood of OVE cows before TZD injection indicated positive energy balance and higher insulin sensitivity. Administration of TZD increased blood concentrations of glucose, insulin, and beta-hydroxybutyrate (BHBA) at 2 to 4 weeks after diet initiation, while the concentration of NEFA and adiponectin (ADIPOQ) remained unchanged during TZD. The TZD upregulated the mRNA expression of *PPARG* and its targets *FASN* and *SREBF1* in SAT, but also *SUMO1* and *UBC9* which encode sumoylation proteins known to down-regulate *PPARG* expression and curtail adipogenesis. Therefore, a post-translational response to control *PPARG* gene expression in SAT could be a counteregulatory mechanism to restrain adipogenesis. The OVE cows had greater expression of the insulin sensitivity-related genes *IRS1*, *SLC2A4*, *INSR*, *SCD*, *INSIG1*, *DGAT2*, and *ADIPOQ* in SAT. In skeletal muscle, where *PPARA* and its targets orchestrate carbohydrate metabolism and fatty acid oxidation, the OVE cows had greater glyceroneogenesis (higher mRNA expression of *PC* and *PCK1*), whereas CON cows had greater glucose transport (*SLC2A4*). Administration of TZD increased triacylglycerol concentration and altered expression of carbohydrate- and fatty acid oxidation-related genes in skeletal muscle. Results indicate that overfeeding did not affect insulin sensitivity in nonpregnant, nonlactating dairy cows. The bovine PPARG receptor appears TZD-responsive, with its activation potentially leading to greater adipogenesis and lipogenesis in SAT, while differentially regulating glucose homeostasis and fatty acid oxidation in skeletal muscle. Targeting PPARG via dietary nutraceuticals while avoiding excessive fat deposition might improve insulin sensitivity in dairy cows during times such as the peripartal period when the onset of lactation naturally decreases systemic insulin release and sensitivity in tissues such as AT.

## Introduction

In non-ruminants, the peroxisome proliferator-activated receptors (**PPAR**) play a crucial role in the regulation of fatty acid metabolism and inflammatory responses [1]. The PPAR family is composed of three subtypes: PPAR-gamma (**PPARG**), PPAR-alpha (**PPARA**), and PPARδ/β (**PPARD**), among which the expression of PPARG and PPARA subtypes are associated with adipogenesis/lipogenesis and catabolism of fatty acids, respectively [1]. A comprehensive review in ruminants underscored the potential for manipulating PPAR to fine-tune metabolism and immune function during growth and lactation [2]. Because PPARG plays a pivotal role in adipogenesis of adipose tissue (**AT**), its regulation through insulin-sensitizing agonists, nutrition, and obesity in dairy cows could have important implications in the control of energy homeostasis, particularly during the period between late pregnancy and early lactation, which is characterized by marked AT lipolysis due in part to a reduction in insulin concentration. This is one of several adaptations in dairy cows to prioritize the use of nutrients for mammary synthesis of milk.

Thiazolidinediones are potent insulin-sensitizing agonists of the nuclear receptor PPARG [3]. The results of previous studies with dairy cows underscored the possibility that 2,4-Thiazolidinedione (**TZD**) can ameliorate insulin insensitivity, likely via PPARG [4–6]. The administration of TZD (4 mg of TZD/kg of BW) during the late prepartum period appeared to alter the dynamics of plasma glucose, nonesterified fatty acids (NEFA), and beta-hydroxybutyrate (BHBA) concentrations as well as dry matter intake (DMI) during the periparturient period, and helped cows maintain body condition score (BCS) postpartum [4, 7]. Furthermore, a greater concentration of insulin in blood was observed in TZD-treated cows, which likely accounted for the lower NEFA [6]. In humans, the mean clearance rate (i.e. change in plasma concentration over time) of rosiglitazone, a TZD, was reported to be lower in female compared with male patients of the same body weight [8]. However, no such data exist for dairy cow but could be key for understanding temporal changes in profiles of PPAR-related and insulin-sensitivity related genes [2].

Our hypothesis was that plane of nutrition leads to changes in BW and insulin sensitivity in subcutaneous AT (**SAT**) and skeletal muscle tissue. Furthermore, the peripheral and systemic insulin-sensitizing effect of TZD occurs at least in part via molecular alterations in PPAR signaling networks. Therefore, the objective of the current study was to evaluate the effect of plane of energy nutrition and TZD administration on energy intake (NE_L_), body mass, body fatness, energy balance markers (e.g. NEFA, BHBA, and glucose), insulin, and the insulin-sensitizing hormone adiponectin (**ADIPOQ**) in non-pregnant and non-lactating dairy cows.

## Materials and Methods

### Experimental Design, Diet and Animal Management

The Institutional Animal Care and Use Committee (IACUC) of the University of Illinois approved all procedures for this study (protocol #12134). Fourteen nonlactating non-pregnant Holstein cows [initial body weight (BW, kg) = 717 ±39; initial BCS = 3.31 ± 0.14] were assigned randomly into the treatment groups. Cows were offered the TMR once daily at 0600 h and had unlimited access to fresh water. All cows were fed a control diet (low-energy, CON; NE_L_ = 1.30 Mcal/kg) to meet 100% of National Research Council (NRC) requirements at *ad libitum* DMI for 3 weeks, after which half of the cows were assigned to a higher-energy diet (OVE; NE_L_ = 1.60 Mcal/kg) and half of the cows continued on CON for 6 weeks. The OVE diet was fed *ad libitum* and resulted in cows consuming ~180% of NRC requirements. Control cows were fed to consume only 100% of NRC. The ingredient and nutrient composition of both diets are presented in Table 1. All cows received an intravenous injection of 4 mg TZD/kg of BW daily into the jugular vein starting 2 weeks after the initiation of treatments and for 2 additional weeks. The last 2 weeks of the study served as the washout period. Cows were housed in ventilated indoor pens (10 m × 15 m; photoperiod of 8 h light and 16 h dark) equipped with individual electronic transmission gates and transponders (American Calan, Northwood, NH) for access to feed. Each pen had 10 sand-bedded free stalls with at least one stall per cow.

**Table 1:** Ingredient and analyzed nutrient composition of moderate energy (OVE) and low energy (CON) diets (% of DM).

Component	Diet	
Ingredient, % of DM	CON	OVE
Alfalfa hay	2.00	5.97
Alfalfa silage	8.88	13.61
Ground shelled corn	4.04	12.56
Corn silage	33.21	54.08
Dicalcium phosphate	0.79	0.70
Limestone	0.82	0.84
Magnesium chloride	0.46	0.70
Magnesium oxide	0.40	0.38
Magnesium sulfate	0.99	1.05
Mineral—vitamin premix^1^	0.20	0.21
Salt	0.20	0.14
Soybean meal, 48% CP	11.56	4.35
Urea	0.20	0.19
Vitamin A^2^	0.01	0.01
Vitamin D^3^	0.01	0.01
Vitamin E^4^	0.26	0.24
Wheat straw	35.97	-
Whole cottonseed	-	4.98
Chemical Analysis		
DM of diet, %	42.40	37.80
NE_L_, Mcal/kg	1.30	1.60
CP, % DM	14.08	14.45
ADF, % DM	34.40	26.30
NDF, % DM	50.40	38.30

^1^Contained a minimum of 5% Mg, 10% S, 7.5% K, 2.0% Fe, 3.0% Zn, 3.0% Mn, 5,000 mg/kg of Cu, 250 mg/kg of I, 40 mg/kg of Co, 150 mg/kg of Se, 2,200 kIU/kg of vitamin A, 660 kIU/kg of vitamin D3, and 7,700 IU/kg of vitamin E.

^2^Contained 30,000 kIU/kg.

^3^Contained 5,009 kIU/kg.

^4^Contained 44,000 IU/kg.

### Sample Collection

Blood samples were collected before the morning feeding from the coccygeal vein or artery every 5 d ± 2 from –7 to 14 d relative to diet initiation, before TZD administration or from 15 to 28 d relative to diet initiation and during TZD administration for measurement of metabolites and hormones. Samples were collected into evacuated tubes (Vacutainer, BD and Co., Franklin Lakes, NJ) containing clot activator or lithium heparin. After blood collection, tubes containing lithium heparin were placed on ice, while the tubes with clot activator were kept ~ 30 min at 21°C until centrifugation. Serum and plasma were obtained by centrifugation of clot activator and lithium heparin tubes, respectively, at 1,900 × *g* for 15 min and frozen at −80°C until later analysis.

#### Blood Metabolites and Hormones

Blood metabolites were analyzed in lithium heparin samples at 37°C following the procedures previously described by Bionaz et al. [9] in a clinical auto-analyzer (ILAB 600, Instrumentation Laboratory, Lexington, MA, USA). Glucose, NEFA and BHBA were determined using commercial kits purchased from Instrumentation Laboratory (IL Test), Wako Chemicals GmbH (Neuss, Germany) and Randox Laboratories Ltd. (Crumlin, Co. Antrium, UK) respectively. Adiponectin was measured in serum samples using the ELISA assay developed by Mielenz et al. [10], while insulin was assayed by a double-antibody radioimmunoassay (RIA) using a primary antiserum to bovine insulin as described by Sosa et al. [11]. The ratios of NEFA and glucose to insulin were calculated to estimate systemic insulin sensitivity.

### Tissue biopsies, RNA Isolation, Primer Design and Evaluation, and Quantitative PCR

Subcutaneous AT and skeletal muscle biopsies were collected from alternate sides before the morning feeding at 14, 21 and 28 d, and 35 d relative to diet initiation. The TZD was injected starting on d 14 and continued for 2 additional weeks. Details of these procedures are reported in the supporting information. The selection of the primers for SAT was conducted based on core cellular functions, e.g. insulin signaling and responsiveness, adipogenic and lipogenic enzymes/inducers, and post-translational modifiers; while the focus on skeletal muscle tissue was based on core cellular functions, e.g. lipid and carbohydrate biosynthesis and metabolism (Table A and Table B in S1 File). The amplicons were sequenced and the fragment sequences were blasted and confirmed using the National Center for Biotechnology Information (NCBI). The sequences are presented in the supporting informationhttp://www.journalofdairyscience.org/. The geometric mean of the internal control (ICG) genes *GAPDH*, ribosomal protein S9 (*RPS9*), and ubiquitously-expressed transcript (*UXT*) were used for data normalization (V2/3 ≤0.15) in SAT, while the geometric mean of reference genes mitochondrial ribosome-associated GTPase 1 (*MTG1*), ribosomal protein S15A (*RPS15A*) and *UXT* were used for data normalization (V2/3 ≤0.15) in skeletal muscle tissue.

### Muscle Triacylglycerol Concentration

A total of 50 mg of tissue was first homogenized in 1.5 mL of PBS/10 mM EDTA using a hand-held homogenizer (Tissue-Tearor, Biospec Products). Subsequently, 200 μL of GPBS-EDTA along with 3 mL of isopropanol-hexane-water (80:20:2 vol/vol) were added to each sample and the mixture incubated covered with aluminum foil for 30 min at room temperature. One mL of hexane-diethyl ether (1:1) was then added to each sample followed by vortexing and incubating for 10 min at room temperature (protected from light). One mL of water was added to each sample to separate the lipid phase and the mixture was vortexed. Samples were incubated covered with aluminum foil for ~20 min at room temperature. The organic phase was then aspirated and placed into glass vials, prior to evaporation under a stream of N gas. An 8-point TAG standard was prepared with Infinity TG reagent (cat#10010509, Cayman Chemicals). Each sample was mixed with 540 μL of Infinity TG reagent prior to vortexing. A total of 160 μL of this sample mixture was pipetted into a flat-bottom 96-well plastic microplate. The plate was incubated for 15 min at 37°C prior to determining absorbance at 540 nm using a microplate reader. Concentration of TAG was calculated from the standard curve. The final concentration is expressed as mg TAG/g of tissue.

### Western Blot Analysis

The entire procedure is reported in the supporting information. Briefly, Western blotting was conducted to verify a subset of the key changes observed in protein expression of PPARG in SAT. The SAT was homogenized and hundred micrograms of total protein was subjected to 12% SDS/PAGE and transferred to a PVDF membrane (Millipore). The membrane was probed first with rabbit anti- PPARG antibody (Abcam, Cambridge, MA) and rabbit anti-GAPDH antibody (Abcam). The secondary antibody was goat polyclonal secondary antibody to rabbit IgG—H&L (HRP) (Abcam). Immunodetection using the Clarity Western ECL Substrate (Bio-rad, Hercules, CA) was performed according to the manufacturer’s instruction. The intensities of the bands were quantified [12, 13] using ImageJ software [14] and the expression of PPARG protein was normalized to the expression level of GAPDH protein.

### TZD Measurement in Serum

Two hundred μL of serum was precipitated by addition of 200 μL of ethyl acetate in 2 mL micro centrifuge tubes. The tubes were vortexed for 2 min and centrifuged at 10,000 g for 10 min. The supernatant was transferred to a clean tube. All extracts were evaporated under nitrogen at room temperature. Then they were reconstituted with 100 μL of ethanol [15]. The HPLC system included a Hewllett Packard 1100 System with diode array detector. Separation were performed on a Hypersil BDS C-18 (250 mm x 4.6 mm i.d., 5 μm particle size) (Thermo Scientific, Waltham, MA) analytical column. The TZD was eluted isocratically with methanol-20 mM MOPS buffer at pH 7.4 (80:20, v/v) at a flow rate of 1 mL min-1, with TZD detection at 230 nm. In all cases, a 20 μL aliquot was injected by duplicate into the HPLC system [16].

### Gene Network Analysis

Gene network analysis was performed using Ingenuity Pathway Analysis^®^ (IPA; http://www.ingenuity.com, Redwood City, CA). Only direct interactions among the genes analyzed in the present study were included in the analyses. The IPA database considers only the known interactions in published studies with humans and rodents. The data obtained on biopsies harvested at 14 d of diet initiation were considered as the reference point to generate the IPA input data as fold-change. Gene networks generated from the analysis can be found in the S1 File of the Supporting Information material.

### Statistical Analysis

All data were analyzed with the Proc MIXED procedure of SAS 9.4 (SAS Institute Inc., Cary, NC). After normalization with the geometric mean of the ICG, the triplicate qPCR data for each gene were averaged and then log_2_ transformed prior to statistical analysis. Fixed effects in the model were diet, time, and diet × time. Cow within diet was designated as the random effect. Initial BW and BCS were included as covariates in the analysis for all variables, except when the covariate was not significant (*P >* 0.05). The Kenward-Roger statement was used for computing the denominator degrees of freedom, while spatial power was used as the covariance structure. The same statistical model was used for the protein data. Spearman rank correlation coefficients were derived to identify possible relationships among the interval-measured blood variables. Significance was declared at *P* ≤ 0.05, while trends were declared at *P* ≤ 0.10 for the gene expression data.

## Results

### Energy Intake and Body Mass

**Fig 1:**
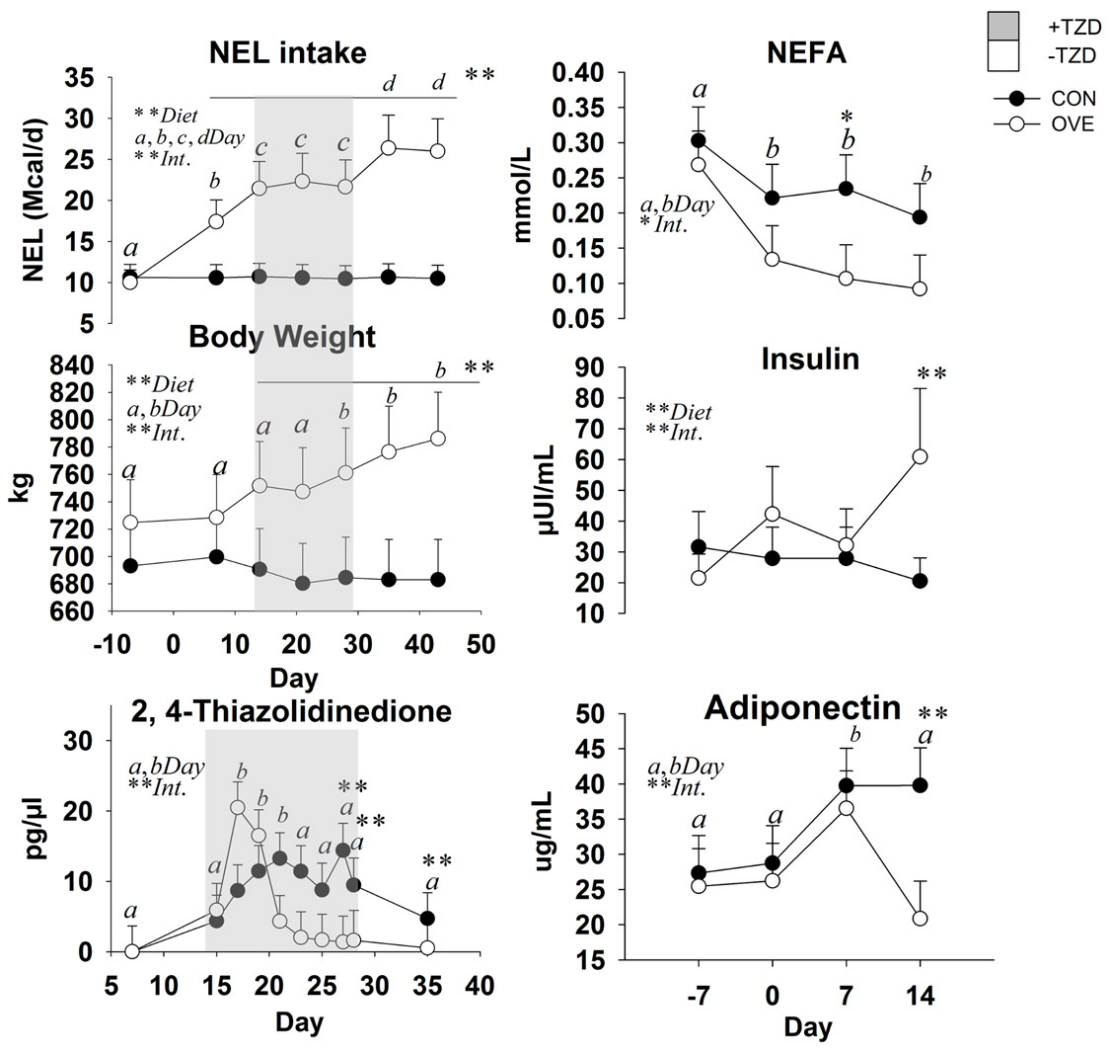
Daily NEL (Mcal) intake, weekly body weight, temporal concentrations of serum 2,4-Thiazolidinedione (–7 to 42 d), and temporal concentrations (–7 to 14 d relative to diet initiation; before TZD administration) of serum NEFA, insulin and adiponectin in cows fed either a low-energy diet (CON, 1.30 Mcal/kg; n = 7) or high-energy diet (OVE, 1.60 Mcal/kg; n = 7) before, during (i.e. gray area) and after (washout) TZD administration. Significant differences between diet, day and diet × day (Int., *P* ≤ 0.05) are denoted with ** or a,b.

Cows fed OVE had greater NE_L_ intake (*P* < 0.05; 19.91 *vs*. 10.58 Mcal/d; diet × day *P* < 0.05; Fig 1) than CON cows. Body weight was greater (diet × day *P* < 0.05; Fig 1) in OVE compared with CON cows at 2 to 6 weeks after diet initiation. The NE_L_ intake and BW were affected by the interaction of diet × day (*P* < 0.05) because of the gradual increase of DMI in OVE cows compared with the constant NE_L_ intake in CON cows. The TZD administration did not affect NE_L_ intake and BW (Fig 1) but OVE cows had greater energy intake (*P* ≤ 0.05) during the washout period compared with the TZD administration period at 28 d.

### Blood Metabolites and Hormones before TZD Administration

Main effects of diet, time, and their interactions for biomarkers are reported in Fig 1. The OVE cows had lower concentrations of NEFA (diet × day, *P* ≤ 0.05; Fig 1) and ADIPOQ (diet × day, *P* ≤ 0.05; Fig 1) compared with CON. There was an increase (diet × day, *P* ≤ 0.05; Fig 1) in the concentration of insulin in OVE cows at 2 weeks after diet initiation, while there was no effect on glucose and BHBA. The ratio of glucose and NEFA to insulin was affected by the interaction of diet × day (*P* ≤ 0.05) because of the gradual decrease in OVE at 14 d (Table 2).

**Table 2:** Glucose and NEFA to insulin ratios in nonlactating dairy cows from –7 to 14 d relative to diet initiation, before TZD administration or from 15 to 28 d relative to diet initiation and during TZD administration in cows fed either controlled-energy diet (CON, 1.30 Mcal/kg; n = 7) or moderate-energy diet (OVE, 1.60 Mcal/kg; n = 7) during TZD administration. Values are LSM ± SEM.

	Diet			Day					*P*-value		
Item	CON	OVE	SEM	-7	0	7	14	SEM	Diet	Day	Diet × Day
**Before TZD**											
Glucose/insulin	0.59	0.53	0.03	0.59	0.54	0.57	0.53	0.04	0.05	0.18	< 0.01
NEFA/insulin	0.23	0.17	0.03	0.24^a^	0.19^b^	0.20^b^	0.18^b^	< 0.01	< 0.01	< 0.01	< 0.01
	Diet					Day			*P*-value		
**During TZD**	CON	OVE	SEM	15	20	25	28	SEM	Diet	TZD	Diet × TZD
Glucose/insulin	0.59	0.51	0.03	0.58^a^	0.51^b^	0.54^b^	0.54^b^	0.04	< 0.01	0.08	0.68
NEFA/insulin	0.23	0.16	0.03	0.22^a^	0.18^b^	0.19^a^	0.18^b^	0.02	< 0.01	0.02	0.32

^a,b^ *diet*, Day, TZD, *diet* × Day and *diet* × TZD within a row with different superscripts differ (*P* ≤ 0.05).

### Blood Metabolites and Hormones during TZD Administration

In OVE as well as CON cows, TZD administration increased the concentrations of insulin, glucose, and BHBA (Table 3). The concentrations of insulin and BHBA were higher (*P* ≤ 0.05), and concentrations of NEFA and ADIPOQ lower in OVE compared with CON cows (Table 3). The TZD did not affect (*P* > 0.10) the serum concentrations of NEFA and ADIPOQ (Table 3). The ratio of glucose and NEFA to insulin decreased (*P* < 0.05) in OVE compared with CON, or in response to TZD (Table 2).

**Table 3:** Concentration of blood metabolites from 15 to 28 d relative to diet initiation for cows either fed controlled-energy diet (CON, 1.30 Mcal/kg; n = 7) or moderate-energy diet (OVE, 1.60 Mcal/kg; n = 7) during TZD administration. Values are LSM ± SEM.

	Diet			Day					*P*-value	
Item	CON	OVE	SEM	15	20	25	28	SEM	Diet	TZD
**During TZD**										
Glucose (mmol/L)	4.49	4.66	0.07	4.55^c^	4.51^c^	4.65^d^	4.61^c^ ^d^	0.06	0.16	0.10
Insulin (μU/mL)	26.50	44.55	7.64	27.10^c^	41.57^d^	35.43^c^ ^d^	34.90^c^ ^d^	8.89	< 0.01	0.10
NEFA (mmol/L)	0.63	0.49	0.06	0.18	0.14	0.15	0.11	0.05	0.02	0.20
BHBA (mmol/L)	0.23	0.40	0.02	0.27^a^	0.32^a^	0.37^b^	0.31^a^	0.03	< 0.01	0.04
ADIPOQ (μg/mL)	35.72	30.93	1.80	34.63	33.70	30.27	34.72	2.18	0.08	0.40

^a,b^ Means within a row with different superscripts differ (*P* ≤ 0.05) for TZD, while ^c,d^ the trend differs at (*P* ≤ 0.10). The Diet × Day interaction was not significantly affected.

### TZD Concentration in Blood

The main effects of diet, time, and interaction on serum TZD are reported in Fig 1. An interaction (diet × day, *P* ≤ 0.05) was observed because of a lower blood TZD concentration in OVE at 18 d post-injection and for CON at 20 d post-injection. Those responses were followed by lower blood TZD concentration in both treatment groups from d 20 to 35. The concentration of TZD in blood was lower in OVE compared with CON over time. There was no overall effect (*P* > 0.05) of diet on TZD concentration in blood (Fig 1).

### Gene Expression

#### Subcutaneous Adipose Tissue

**Fig 2:**
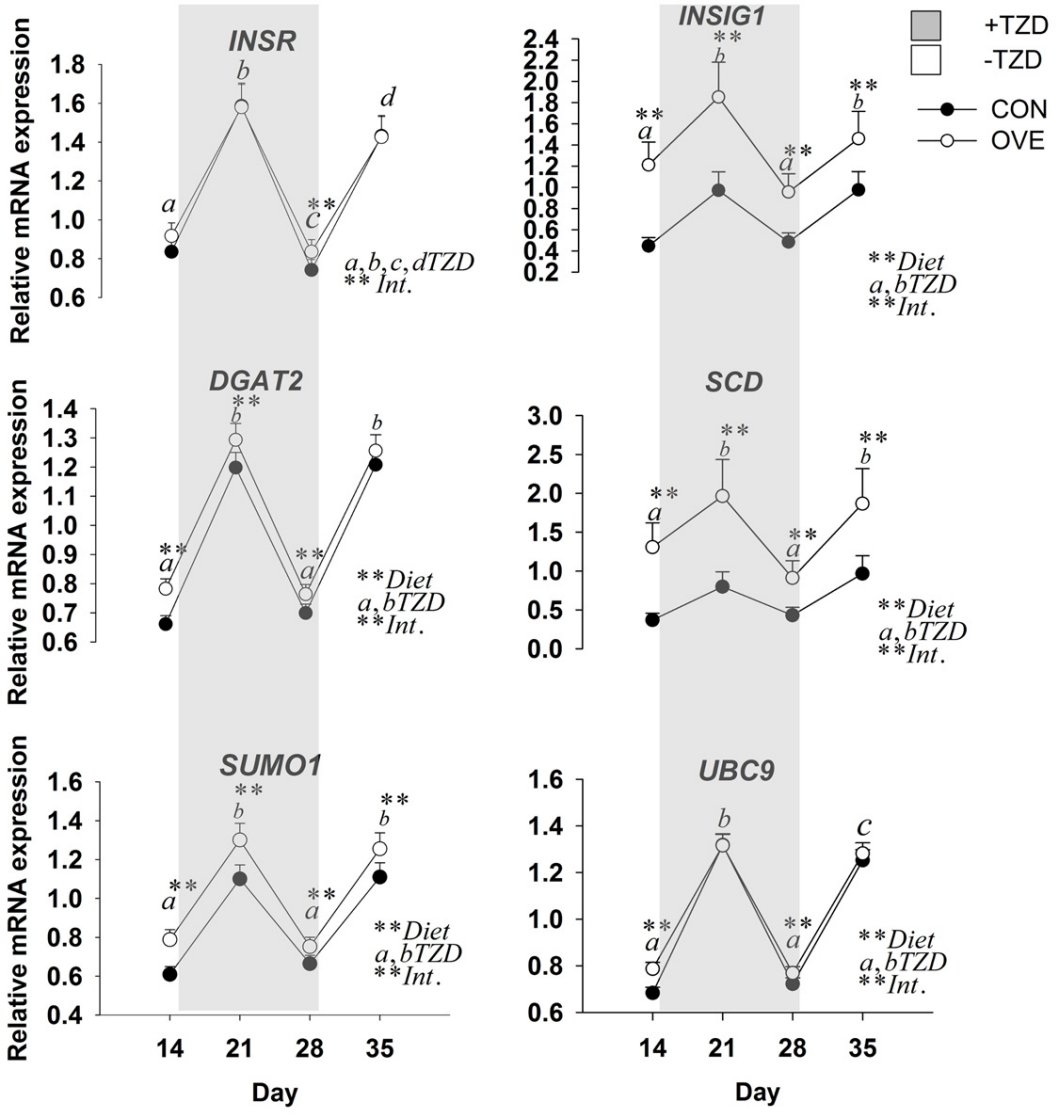
Expression of insulin signaling (*INSR*), lipogenic enzymes/inducers (*DGAT2*, *INSIG1* and *SCD*) and post-translational modifiers (*SUMO1*, *UBC9*) in subcutaneous adipose tissue of cows fed either a low-energy diet (CON, 1.30 Mcal/kg; n = 7) or high-energy diet (OVE, 1.60 Mcal/kg; n = 7) before, during (i.e. gray area) and after (washout) TZD administration. Significant differences between diet, TZD and diet × TZD (Int., *P* ≤ 0.05) are denoted with ** or a,b,c,d.

The main effects of diet, TZD, and interactions for the genes in SAT are reported in Fig 2 and Table 4. The mRNA expression of *SLC2A4*, *ADIPOQ*, *FASN*, *DGAT2*, *INSIG1*, *SUMO1*, and *UBC9* was overall greater (diet, *P* < 0.05) in OVE compared with CON cows (Fig 2; Table 4). The mRNA expression of insulin signaling (*INSR*, *IRS1*, and *SLC2A4*), lipogenic enzymes/inducers (*SCD*, *INSIG1*, and *DGAT2*), post-translational modifiers (*SUMO1*, and *UBC9*), and *ADIPOQ* increased (*P* ≤ 0.05) at 1 weeks after TZD administration, but subsequently decreased (*P* ≤ 0.05) at 2 weeks post-TZD administration. Their expression increased again to values observed after 1 week of TZD at the end of the washout period (Fig 2; Table 4).

**Table 4:** mRNA expression of insulin responsive genes in adipose tissue and skeletal muscle, and triacylglycerol (TAG) content in skeletal muscle (mg TAG/g of tissue) at 14 to 28 d relative to diet initiation and after a 7-d washout period (35 d) in cows fed a controlled-energy diet (CON, 1.30 Mcal/kg; n = 7) or a moderate-energy diet (OVE, 1.60 Mcal/kg; n = 7). All cows received an intravenous injection of TZD (+) for 2 weeks after biopsy collection, at 14 d after initiation of dietary treatments, with the additional 2 weeks of study serving as a washout period. Values are LSM ± SEM.

	Diet			Day					*P*-value	
Gene	CON	OVE	SEM	14	21	28	35	SEM	Diet	TZD
**TZD**				-	+	+	-			
**Adipose**										
*IRS1*	1.02	1.10	0.06	1.07^a^	1.11^b^	1.03^a^	1.04^a^	0.05	0.11	< 0.01
*SREBF1*	1.46	1.61	0.12	1.75^a^	1.53^b^	1.65^a^	1.27^c^	0.11	0.12	< 0.01
*PPARG*	1.46	1.57	0.15	2.08^a^	1.24^b^	1.89^c^	1.08^d^	0.10	0.35	< 0.01
*FASN*	0.94	1.92	0.29	2.08^a^	0.97^b^	1.74^c^	0.92^b^	0.24	< 0.01	< 0.01
*SLC2A4*	1.00	1.48	0.19	1.01^a^	1.73^b^	0.83^c^	1.49^d^	0.16	< 0.01	< 0.01
*ADIPOQ*	0.90	1.21	0.18	0.77^a^	1.60^b^	0.68^a^	1.41^b^	0.16	0.03	< 0.01
**Muscle**										
*IRS1*	0.99	0.94	0.06	1.05^a^	0.95^a^	0.91^b^	-^1^	0.06	0.31	< 0.01
*PPARA*	1.00	1.00	0.05	1.03^a^	1.02^a^	0.97^b^	-	0.04	0.93	< 0.01
*ACADVL*	1.00	1.01	0.04	1.02^a^	1.01^a^	0.98^b^	-	0.03	0.58	0.02
*PDK4*	1.02	0.85	0.09	0.98^a^	0.85^b^	0.95^a^	-	0.09	0.02	0.03
*CPT1A*	1.07	0.95	0.04	1.01^a^ ^b^	0.96^a^	1.05^b^	-	0.04	< 0.01	< 0.01
TAG (mg/g tissue)	41.09	42.28	4.52	28.66^a^	43.70^b^	43.31^b^	51.06^c^	4.83	0.85	< 0.01

^a,b,c,d^ *diet*, TZD within a row with different superscripts differ (*P* ≤ 0.05).

^1^Tissue sample not available.

Despite the up-regulation of insulin sensitivity-related genes, the mRNA expression of the adipogenic gene *PPARG* and its lipogenic target *FASN* as well as the insulin responsive transcription regulator *SREBF1* decreased at 1 week after TZD administration, and subsequently increased (*P* ≤ 0.05) at 2 weeks post TZD administration. Opposite to the response detected for insulin signaling and post-translational modifiers, the expression of *PPARG*, *FASN*, and *SREBF1* decreased again at the end of the washout period (Fig 2; Table 4).

#### Skeletal Muscle Tissue

**Fig 3:**
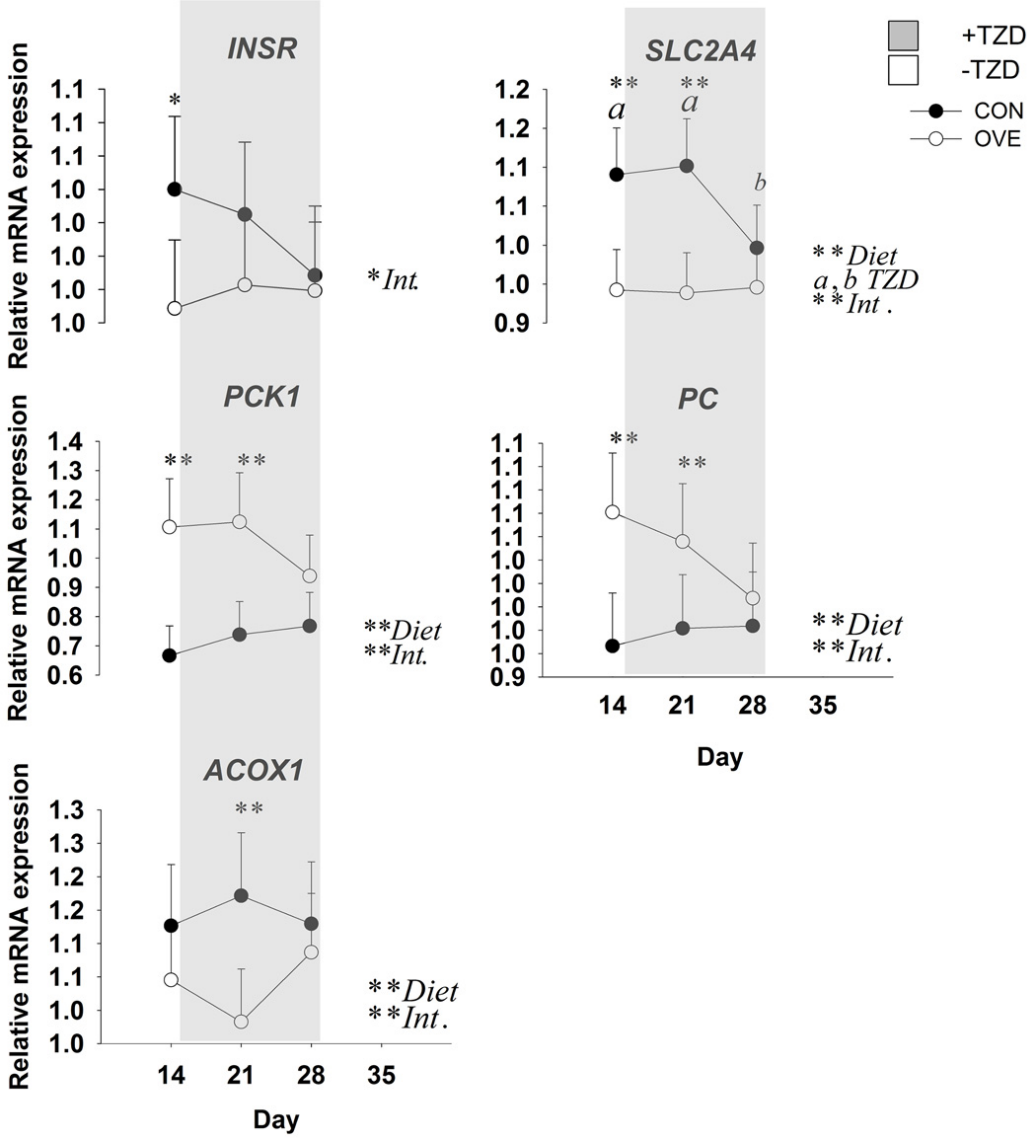
Expression of insulin signaling (*SLC2A4* and *INSR*), fatty acid oxidation (*ACOX1*), glucose homeostasis (*PC* and PCK1) in skeletal muscle tissue of cows fed either a low-energy diet (CON, 1.30 Mcal/kg; n = 7) or high-energy diet (OVE, 1.60 Mcal/kg; n = 7) before, during (i.e. gray area) and after (washout) TZD administration. Significant differences between diet, TZD and diet × TZD (Int., *P* ≤ 0.05) are denoted ** or a,b, while trends (*P* ≤ 0.10) are denoted with *.

The main effects of diet, TZD, and interactions for the genes in skeletal muscle tissue before and during TZD administration are reported in Fig 3 and Table 4. The OVE cows had lower overall mRNA expression of *PDK4* (Table 4), and its expression decreased at 1 week after TZD administration. Expression of *PDK4* subsequently returned to the same values detected on d 14 at the end of the TZD treatment. The mRNA expression of insulin signaling (*SLC2A4*) and fatty acid oxidation genes (*PPARA* and *ACADVL*) was greater (*P* ≤ 0.05) at 2 weeks after diet initiation and 1 week after TZD administration compared with 2 weeks after TZD administration (Fig 3; Table 4). The OVE cows had a lower (*P* < 0.05) overall mRNA expression of *CPT1A* and *ACOX1* compared with CON cows. The main effect of diet and interaction were due to the lower mRNA expression of *ACOX1* in OVE cows on d 21 (Fig 3). Regardless of diet, TZD administration resulted in modest up-regulation (*P* ≤ 0.05) of *CPT1A* at 2 weeks post-TZD (Table 4). Compared with CON cows, OVE cows had greater (*P* < 0.05) mRNA expression of the glyceroneogenesis-related genes *PCK1* and *PC*. An interaction (diet × day, *P* ≤ 0.05) for the mRNA expression of *PCK1* and *PC* was observed because of a decrease in mRNA expression of both genes in OVE cows at 28 d, 2 weeks after the TZD administration (Fig 3). No effect (*P* > 0.10) of diet, TZD or the interaction was observed for *PPARD* and *DGAT1*.

### Skeletal Muscle Tissue Triacylglycerol Concentrations

The main effect of TZD was due to the greater triacylglycerol (TAG) synthesis during the TZD administration period and washout period at d 21, 28 and 35 (*P* ≤ 0.08; Table 4). No effect (*P* > 0.10) of diet or the interaction was observed for TAG in skeletal muscle.

### Protein Expression in SAT

**Fig 4:**
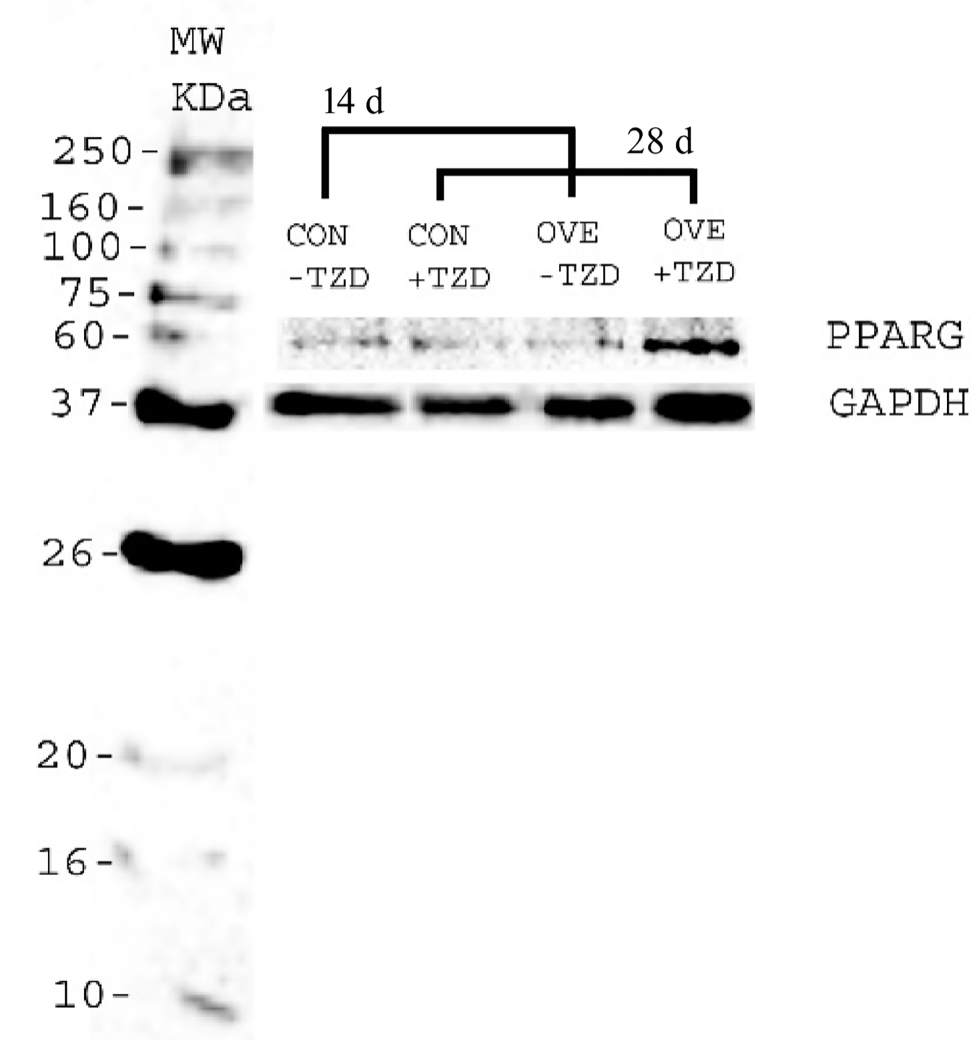
Example of a Western blot of PPARG (58 kDa) and GAPDH (38 kDa) protein from adipose tissue of 2 cows fed either a control diet (CON, 1.30 Mcal/kg; n = 7) or overfed (OVE, 1.60 Mcal/kg; n = 7) at 14 (before TZD injection; -TZD) and 28 d (end of TZD injection; +TZD) relative to diet initiation.

The protein expression of PPARG was numerically greater in overfed cows (diet, *P* = 0.16) (Fig 4). Raw expression data can be found in the S2 File of the Supporting Information material.

## Discussion

Studies using euglycemic clamps [17] and glucose tolerance tests (**GTT**) [5] demonstrated that late-pregnancy in sheep and cows is characterized by altered insulin sensitivity. Decreased insulin sensitivity prepartum and early postpartum contributes to the typical increase in circulating NEFA and it has been speculated that such response is one of the causes for the decrease in voluntary DMI after calving [18, 19]. Previous data from our group provided evidence that overfeeding a higher-energy diet during the last ~20 to 30 d prepartum did not impair SAT insulin signal transduction but resulted in greater NEFA postpartum [20]. The higher NEFA and lower DMI relationship postpartum is associated with several periparturient health disorders, e.g. displaced abomasum, ketosis, fatty liver, retained placenta, metritis, and mastitis. The administration of TZD (4 mg of TZD/kg of BW) during the late prepartum period led to greater concentration of blood insulin the first week postpartum [6], which likely accounted for the lower NEFA, i.e. the drug probably induced greater insulin sensitivity in AT [7].

The nuclear receptor PPARG is central for the maintenance of adipogenesis, hence, targeting this receptor during the late prepartum period has the potential to improve metabolic health and DMI of periparturient dairy cows [2]. In order to study in more detail the mechanistic regulation of insulin sensitivity at the molecular and whole-animal level we utilized non-lactating and non-pregnant dairy cows fed to meet energy requirements or allowed to consume energy ad-libitum in comparison with cows limited to near their energy requirements. The experimental period used was approximately the length of a typical nonlactating period with the caveat that the responses of non-pregnant cows to higher dietary energy might differ from pregnant cows due to nutrient requirements and endocrine environment [21].

### Energy Intake Response

The greater NE_L_ intake throughout the study in cows fed the OVE diet reinforces other observations that dairy cows do not control energy intake or that the set-points above which voluntary energy intake reaches a limit were not attained. Consequently, excess dietary energy intake resulted in surplus that accumulated in fat depots as we reported recently [21]. Two comprehensive reviews underscored that regulation of DMI in dairy cow occurs through a variety of mechanisms including hepatic oxidation of fuels as well as signals reaching brain centers regulating satiety [18, 22]. The results of a study conducted by Smith et al. [4] revealed that administration of 4 mg TZD/kg of BW once daily from 21 d prepartum to parturition increased DMI only during d -7 through +7. The fact that NE_L_ intake in the current study remained the same during TZD administration and gradually increased in OVE cows during the washout period partly agrees with results from Smith et al. [4]. At a mechanistic level, an effect of PPARG agonists on brain centers regulating satiety cannot be discounted because PPARG activation by TZD increased feed intake in rodents without changing hypothalamic neuropeptide Y gene expression [23, 24]. The effect of TZD on bovine hypothalamic genes remains to be determined.

The lower NEFA and higher insulin concentrations over time before TZD administration in OVE cows indicated that energy balance was positive and insulin sensitivity was normal. These results agree with data from late-pregnant cows overfed energy [20]. According to Allen et al. [22] insulin might affect voluntary DMI indirectly through increasing hepatic oxidation of primary and non-primary gluconeogenic precursors (e.g., propionate and butyrate). Such response would occur by increasing the clearance of fuels from the blood, and by decreasing AT lipolysis and supply of NEFA to the liver. In our study, greater insulin in OVE cows did not seem to suppress the NE_L_ intake. Therefore, our data highlight that voluntary feed intake is regulated by multiple factors with alternative mechanisms of action [22].

The unchanged blood concentration of glucose along with greater concentration of insulin before TZD administration in OVE cows was expected. There were positive correlations between NEFA and glucose, and between insulin and BHBA (Table 5). A GTT indicated that in dairy cows fed high- and low-energy diets glucose concentration does not differ greatly [5]. Therefore, the effect of diet on blood glucose clearance and metabolism in the present study was minimal despite elevated concentration of insulin in the OVE cows. The observed responses in glucose and BHBA during TZD agree with previous results [6, 25]. Positive correlations were detected between glucose and insulin, BHBA and insulin before and during TZD administration, and between BHBA and glucose. In contrast, insulin had a positive and NEFA a negative correlation with BHBA (Table 5). The positive correlation between the BHBA and insulin before and during TZD administration likely was due to greater rumen butyrate production as a consequence of greater DMI in OVE cows. The results of previous studies indicated that in non-ruminants, TZD increases glucose utilization in AT, muscle and liver, while it decreases glucose production [26, 27].

**Table 5:** Spearman rank correlation coefficients for the blood metabolites before and during the TZD administration calculated for all cows (n = 14).

Before TZD administration (-7 through 14 d)				
	BHBA	NEFA	ADIPOQ	Insulin
Glucose	0.20	0.27**	0.12	0.028
BHBA		-0.247	-0.04	0.59**
NEFA			-0.11	-0.22
ADIPOQ				-0.16
During TZD administration (15 through 28 d)				
	BHBA	NEFA	ADIPOQ	Insulin
Glucose	0.40**	-0.02	-0.08	0.47**
BHBA		-0.42**	-0.14	0.52**
NEFA			-0.08	-0.40**
ADIPOQ				-0.15

**P < 0.05

Our results of serum ADIPOQ are in accordance with data from mice in which lower energy intake led to lower white AT mass but increased blood levels of ADIPOQ [28]. While seemingly a paradox, the greater mRNA abundance of *ADIPOQ* in SAT concomitant with lower serum concentration of ADIPOQ in OVE cows might be expected because in obese subjects during and after diet-induced weight loss there was a dissociation between AT mRNA expression and serum levels of ADIPOQ [29]. The lack of association between AT mRNA expression and serum concentration of ADIPOQ, the mechanistic role for ADIPOQ in regulating DMI, and the link between insulin sensitivity and regulation of ADIPOQ in bovine deserve further study.

Circulating ADIPOQ concentration is inversely correlated with body fat content in non-ruminants [30], and our data of increased BW in OVE compared with CON cows supports this relationship. In mice, ADIPOQ has no effect on insulin secretion by islets, but can enhance glucose-stimulated insulin secretion in islets from mice with diet-induced obesity [31]. Our results do not suggest any change in the dynamics of blood glucose concentration before TZD administration. However, at 7 to 14 d (Fig 1) there was a trend for a negative correlation between insulin concentration and ADIPOQ concentration in both treatment groups; this is in accordance with a previous study in which insulin inhibited the expression of ADIPOQ in cultured bovine adipocytes [32].

In non-ruminants, the preponderance of data indicate that the TZD class of PPARG agonists are potent inducers of ADIPOQ expression [33]. In the current study, the expression of *ADIPOQ* and insulin-related genes after 7 d of TZD injection responded in a similar fashion as the serum concentration of ADIPOQ. Furthermore, their expression along with serum ADIPOQ decreased after 14 d of TZD injection. The *ADIPOQ* mRNA and serum response to TZD administration is consistent with the presumed higher insulin sensitivity. Differences in *ADIPOQ* mRNA and serum concentration in our data are in agreement with recent findings in which serum *ADIPOQ* was positively correlated with ADIPOQ protein concentrations [34].

### TZD, PPAR, and Insulin Sensitivity

A recent comprehensive review on PPAR in ruminants highlighted that *PPARA* and *PPARD* are involved in the regulation β-oxidation of long-chain fatty acids in liver and skeletal muscle, and *PPARG* in controlling adipogenesis and insulin sensitivity in AT [2]. The expression of both *PPARA* and *PPARD* increased in liver of ketotic cows, a response ascribed in part to the greater influx of NEFA from lipolysis of AT [35]. Although *PPARD* expression in muscle tissue was undetectable in the present study, the decrease in mRNA expression of *PPARA* along with several targets decreased 2 weeks post TZD administration, which suggests a decrease in fatty acid oxidation and a potential increase in TAG accumulation [36].

The increase in insulin sensitivity in AT via activation of PPARG due to exogenous TZD in non-ruminants is well characterized [3]. However, in bovine, only a few studies with conflicting outcomes support the effect of TZD on *PPARG* expression [5, 37]. Although only a numerically-greater effect of diet on PPARG protein expression was detected in OVE cows, the mRNA expression of *PPARG* was altered during the TZD administration in accordance with Schoenberg et al. [7]. It is possible that TZD restores the insulin sensitivity in bovine through *PPARG* via alternate mechanisms, e.g. switching oxidative metabolism to fat synthesis in addition to the ultimate sequestration of fatty acids into TAG. Furthermore, the degree of insulin responsiveness in the skeletal muscle might be influenced indirectly by simultaneous effects on *PPARG* activation in other tissues such as AT [38].

### TZD, Insulin, and Lipogenic Genes

In humans the half-life of an insulin sensitizer such as rosiglitazone was 3 to 7 h after *i*.*v*. administration [39]. Our gene expression data highlighted that in both dietary groups the daily injection of TZD had an insulin-sensitizing effect in SAT at 21 d relative to diet initiation, but disappeared by d 28 when the last dose of TZD was injected. The results of a study in rodents indicated that the lack of *in vitro* response to TZD after 5 h might have been due to a slow rate of PPARG activation. The slower *in vivo* response in skeletal muscle insulin sensitivity could be due to an indirect effect of PPARG activation in AT. We speculate that the coordination between AT and skeletal muscle exists as an indirect mechanism, whereby the regulation of the insulin-responsive genes in skeletal muscle is influenced as a consequence of PPARG activation in another tissue such as AT. Greater TZD concentration at about 21 d relative to diet initiation in OVE cows might have increased the mRNA abundance of insulin sensitivity-related genes in AT, while greater blood TZD clearance at 28 d in OVE cows might have suppressed the mRNA expression of insulin sensitivity-related genes. The greater TZD response in OVE compared with CON cows might be related to the quantity of deposited AT in OVE cows, partly because the main target organ for TZD in bovine appears to be AT, which contains a greater number of PPARG receptors [2].

Perhaps the clearest evidence of an insulin-sensitizing effect of TZD in SAT was the differential change in mRNA expression of *SREBF1* in parallel with *INSR*, *INSIG1*, *SCD*, *SLC2A4*, and *IRS1* at 21 and 28 d. In obese mice, the AT is able to store additional TAG through the adaptive changes of *INSIG1*, *SREBF1*, and *SCD* [40]. The decreased mRNA expression of these genes at 4 weeks after diet initiation and during TZD administration might be due to selective modulation of PPARG occupancy at target gene promoters in AT. Increased mRNA expression of *SREBF1* is potentially indicative of enhanced lipogenesis in TZD-injected cows, supporting a role of this transcription regulator in bovine SAT as in rodents [41].

Among the genes measured in the present study, *SCD* has been associated with diet-induced changes in insulin sensitivity in rodents. For instance, SCD1 knock-out mice fed a high-carbohydrate or high-fat diet remained lean and insulin sensitive, and had reduced AT lipolytic activity and greater hepatic β-oxidation [42]. Work in bovine revealed that *SCD* expression in SAT increased after beef steers were weaned to a higher-starch diet [43], confirming greater adipogenesis during growth of the animals.

In our study, the increased expression of *SCD* on d 21 after TZD might be linked with glucose metabolism in AT and improved glucose disposal induced by the PPARG agonist. In fact, in the overall context of TAG synthesis, the greater expression of *SCD* and *DGAT2* on d 21 suggests an acute enhancement of insulin sensitivity by the sustained increase in circulating insulin. In male rats, rosiglitazone increased the mRNA expression of *DGAT2*. The return to basal expression for both genes on d 28 might have occurred because of greater blood TZD clearance and/or lack of the free receptors due to daily administration of TZD on a daily dosage. Despite the lack of an interaction effect for mRNA expression of *PPARG*, the upregulation of *PPARG* targets such as *FASN* and *DGAT2* prior to TZD was indicative of greater lipogenesis in OVE cows. The above observations, coupled with the differential expression of *PPARG* target genes, underscore that *PPARG* is a major player in SAT.

### Glyceroneogenesis and Insulin Sensitivity in Skeletal Muscle

Glyceroneogenesis is an important pathway in the recycling of intracellular NEFA towards TAG synthesis. The study of Tordjman et al. [44] highlighted that exogenous TZD improves insulin sensitivity partly through induction of glyceroneogenesis, leading to a decrease in NEFA release from fat cells. The TAG/fatty acid cycle includes local intracellular cycling within the AT and extracellular or systemic recycling, as well as formation of the TAG in liver, AT, and muscle. In our study, the significant increase in TAG during the TZD injection and washout period supports the potential sensitizing effect of TZD on the skeletal muscle, hence, the TZD might have led to greater glucose disposal into TAG. The synthesis of TAG in skeletal muscle of non-ruminants relies heavily on the expression and activity of *PCK1* [45]. Regardless of TZD, the greater expression of *PC* and *PCK1* coupled with lower expression of *PDK4* (which is upregulated during negative energy balance; Bionaz et al. [2]) in OVE compared with CON cows might be linked to generation of glycerol-3-phosphate for TAG synthesis through glyceroneogenesis (rather than glycolysis) utilizing a non-glucose precursor (e.g. lactate). This idea is supported by the greater mRNA expression of *SLC2A4* in cows fed CON, suggesting that the skeletal muscle of these cows transported more glucose not only for glycerol-3-phosphate but also to produce ATP through the TCA cycle. These adaptations also are supported by the greater NEFA concentration in CON cows, which might have provided fatty acids as sources of energy in liver and skeletal muscle.

In ruminants, the greater NEFA surge into plasma after parturition was associated with greater expression of *PPARA* target genes such as *CPT1A*, *ACOX1* and *ACADVL* [46]. Positive correlations between plasma BHBA concentration and *CPT1A* and *ACADVL* in liver of dairy cows were reported [46]. However, in diet-induced obese mice the inhibition of CPT1 activity alleviates insulin resistance [47]. Therefore, in our study the lower mRNA expression of *CPT1A* and *PDK4* in the skeletal muscle of OVE cows offers additional support for greater insulin sensitivity in these cows.

### Post Translational Modifiers: Novel Link with Adipogenesis

Another mechanism of control over PPARG activity involves its SUMOylation, which refers to the reversible covalent attachment of small ubiquitin-like modifier (SUMO) peptides (SUMO-1, -2, -3 in mammals). The process involves an activating enzyme (SAE1/SAE2), a conjugating enzyme (UBC9), and an E3 ligase that typically leads to repression of transcription factors [48]. A comprehensive recent review in non-ruminant species underscored the multiple cross-talks between mRNA biogenesis and SUMO proteins in triggering the adipogenesis-responsive factors such as PPAR and SREBP [49]. The opposite expression of *SUMO1* and *UBC9* compared with *PPARG* and *SREBF1* in the present study seems to agree with non-ruminant data. We speculate that the upregulation of post-translational modifiers during the first 7 d of TZD injection was a response to balance the energy expenditure and fine-tune the metabolic master regulators in an autocrine, endocrine, and paracrine fashion in SAT [48]. The moderately-greater expression of mRNA of *SUMO1* and *UBC9* on d 21 and 28 in OVE compared with CON cows might be due to the role of post-translational modifiers to “brake” the processes of adipogenesis and lipogenesis initiated and sustained by *PPARG* and *SREBF1*. Therefore, the observed effect of TZD on *SUMO1* and *UBC9* would serve as a negative feedback to coordinate adaptive mechanisms of *PPARG* and *SREBF1* to TZD.

## Conclusions

The ad-libitum access to a moderate-energy diet markedly increased the energy balance and up-regulated mRNA expression of lipogenic and insulin-induced glucose transport related genes in AT, suggesting that the additional energy did not cause satiety or alter insulin sensitivity. Level of energy intake caused differential TZD clearance rates during its administration, but the TZD was effective in up-regulating PPARG and several target genes in AT during the first 7 d regardless of diet. The fact that these genes are PPARG-targets in non-ruminants suggests that, similar to non-ruminant species, the bovine *PPARG* receptor in dairy cow also is TZD-responsive. Hence, as suggested in previous studies, targeting PPARG during late-pregnancy through early lactation when insulin concentration and sensitivity decreases might be a practical tool to prevent excessive losses in body condition.

## Supporting Information

S1 File Management and sampling. Tail-head adipose tissue biopsy. Hind-leg muscle biopsy. The TZD preparation. RNA extraction, quality assessment, and cDNA synthesis. Primer design and evaluation. Quantitative PCR (qPCR). Relative mRNA abundance of genes within adipose tissue and muscle tissues. Western blot analysis of PPARG within adipose tissue. **Table A**. GenBank accession number, sequence and amplicon size of primers used to analyze gene expression by quantitative PCR. **Table B**. Gene symbol, gene name, and description of the main biological function and biological processes of the targets analyzed in subcutaneous adipose tissue and muscle. **Table C**. Sequencing results obtained from PCR products. **Table D**. qPCR performance among the genes measured in adipose tissue and muscle. **Figure A**. Summary of potential regulatory network of genes involved in insulin response in subcutaneous adipose tissue. **Figure B**. Summary of Diet × Time effects on regulatory network of genes involved in insulin response in subcutaneous adipose tissue. **Figure C**. Summary of potential regulatory network of genes involved in insulin response in muscle tissue. **Figure D**. Summary of Diet × Time effects on regulatory network of genes involved in insulin response in muscle tissue.
(DOCX)

S2 File Western blot data. (XLSX)
