# Supplementary material for: Insulin Sensitivity in Adipose and Skeletal Muscle Tissue of Dairy Cows in Response to Dietary Energy Level and 2,4-Thiazolidinedione (TZD)
Source: PLoS One. 2015 Nov 16;10(11):e0142633. doi: 10.1371/journal.pone.0142633 (PMC4646636; doi:10.1371/journal.pone.0142633)
Supplement: S1 File — Tail-head adipose tissue biopsy. Hind-leg muscle biopsy. The TZD preparation. RNA extraction, quality assessment, and cDNA synthesis. Primer design and evaluation. Quantitative PCR (qPCR). Relative mRNA abundance of genes within adipose tissue and muscle tissues. Western blot analysis of PPARG within adipose tissue. Table A. GenBank accession number, sequence and amplicon size of primers used to analyze gene expression by quantitative PCR. Table B. Gene symbol, gene name, and description of the main biological function and biological processes of the targets analyzed in subcutaneous adipose tissue and muscle. Table C. Sequencing results obtained from PCR products. Table D. qPCR performance among the genes measured in adipose tissue and muscle. Figure A. Summary of potential regulatory network of genes involved in insulin response in subcutaneous adipose tissue. Figure B. Summary of Diet × Time effects on regulatory network of genes involved in insulin response in subcutaneous adipose tissue. Figure C. Summary of potential regulatory network of genes involved in insulin response in muscle tissue. Figure D. Summary of Diet × Time effects on regulatory network of genes involved in insulin response in muscle tissue. (DOCX) [file pone.0142633.s001.docx]

**S1 File**

**Management and Sampling**

The OVE and CON diets were mixed and fed as TMR. All cows were individually fed once daily at 0600 h. Individual feed offered and feed refusals were recorded daily. Cows had unlimited access to fresh water. All cows were fed a control diet (CON; NE_L_ = 1.30 Mcal/kg) to meet 100% of NRC requirements for 3 wk, after which half of the cows were assigned to a moderate-energy diet (OVE; NE_L_ = 1.60 Mcal/kg) and half of the cows continued on CON for 6 wk. The OVE diet was fed *ad libitum*. Cow BW and BCS were recorded twice weekly for all cows before the morning feeding. Individual feed ingredients were sampled weekly and DM content [[1](#_ENREF_1)] was determined for each component (Table 1). Rations were adjusted for DM of ingredients on a weekly basis. Weekly feed ingredient samples were dried and frozen at −20°C and then composited monthly for analysis of DM, CP, NDF, and ADF by wet chemistry techniques at a commercial laboratory (Dairy One, Ithaca, NY). The NE_L_ value of each feed component (Dairy One) was used to calculate the NE_L_ content of the diet. Net energy intake (NE_L_) was calculated by multiplying the daily DMI by NE_L_ density of the diet determined from monthly composite samples. Net energy for maintenance (NEM) was calculated as BW^0.75^ × 0.08.

**Tail-Head Adipose Tissue Biopsy**

Subcutaneous AT biopsies were collected from alternate sides of the tail-head region before the morning feeding at 14, 21, 28 and 35 d relative to diet initiation. The hair of the surgical area was cut closely with clippers and washed with an iodine disinfectant mixture. Lidocaine-HCl (5 mL; Agri Laboratories) was given intramuscularly to anaesthetize the biopsy area 10 min before performing a ~2-cm incision. Adipose tissue (2–4 g) was collected with scalpel and forceps by blunt dissection. The incision was then closed with surgical staples (Multi-Shot Disposable Skin Stapler, Henry Schein) and antimicrobial iodine ointment was applied to the wound. The wound was carefully monitored for the following 7 d. The tissue was quickly blotted with sterile gauze to remove residual blood and snap-frozen in liquid nitrogen until RNA extraction for gene expression analysis. The wounds and the vital signs were checked on a daily base for 7 days after biopsy to avoid any infection.

**Hind-Leg Muscle Biopsy**

A 2-cm incision was made using a sterile scalpel blade. Approximately 200 mg of tissue was removed using a biopsy needle (Bard Magnum; 12 gauge x 16 cm; C. R. Bard, Inc., Murray Hill, NJ) before the morning feeding at 14, 21 , 28 and 35 d relative to diet initiation. The samples were snap frozen in liquid nitrogen for further analysis. After completing the biopsies, pressure was applied with sterile gauze to stop any external bleeding, the surrounding area was cleansed with sterile saline to remove blood, and the incision was closed with 3 surgical staples. Antimicrobial ointment was applied to the incision site. The wounds and the vital signs were checked on a daily base for 7 days after biopsy to avoid any infection.

# The TZD preparation

# The 2,4-Thiazolidinedione (Cat. Number: 375004-100G, Tech., 90%; Sigma Aldrich, St. Louis, MO) was aliquot in 4.2 g in sterile, autoclaved glass bottles and sealed subsequently. Two hours before administration, 100 mL of physiological saline (0.9% Sodium Chloride Injection, USP, Rockville, MD) was injected into the bottle and placed on a Signature Multi-Tube Vortexer (Cat. Number: 58816-115, VWR, Batavia, IL) at 2000 rpm for 2 h. The 4 mg TZD/kg of BW daily was calculated based on the actual weekly BW and injected into the jugular vein using Scalp Vein Butterfly Sets starting at 2 wk after the initiation of diet for 2 additional wk. The last 2 wk of the study served as the washout period.

**RNA Extraction, Quality Assessment and cDNA synthesis**

Adipose tissue and muscle tissues were weighed (~50 mg and ~200 mg, respectively) and immediately placed into ice-cold QIAzol Lysis Reagent (~1 and 1.2 mL, respectively; Qiagen, Valencia, CA) in a 2 ml RNase/DNase Free tubes with the O-ring, 1 bead (Qiagen: Cat. No: 69989, 5 mm) per tube was added using the Qiagen bead dispenser. The tubes were loaded into a semi-automated homogenizer and the samples were homogenized for two times 30 sec. with 1 min. incubation time on the ice. The Total RNA plus miRNA extraction was performed following the procedure recommended by Qiagen (miRNeasy Mini Kit; Cat. # 217004)*.* The advantage of the kit was to remove the genomic DNA and includ column RNA purification as well. The RNA concentration was measured with NanoDrop ND-1000 spectrophotometer (NanoDrop Technologies). The purity of RNA was assessed by ratio of optical density OD260/280, which were above 1.9 for all samples. The RNA integrity number (RIN) was assessed by electrophoretic analysis of 28S and 18S rRNA subunits using a 2100 Bioanalyzer (Agilent Technologies), and values were above 7 for all samples. A portion of the RNA was diluted to 100 ng/μL with DNase/RNase-free water for cDNA synthesis through reverse transcription PCR. cDNA was synthesized using 100 ng of RNA, 1 μL of Random Hexamer Primers (Cat. #11034731001, Roche, Pleasanton, CA), and 9 μL of DNase/RNase-free water. The mixture was incubated at 65°C for 5 min and kept on ice for 3 min. A total of 9 μL of master mix composed of 4 μL of 5X First-Strand Buffer, 1 μg of dT18 (Integrated DNA Technologies), 2 μL of 10 m*M* dNTP mix (Cat. #18427-088, Invitrogen Corp., Grand Island, NY), 0.25 μL (200 U/µL) of RevertAid Reverse Transcriptase (Cat. # EP0441, Fermentas Inc., Pittsburgh, PA), 0.125 μL (20 U/µL) of RiboLock RNase Inhibitor (Cat. #EO0381, Fermentas Inc., PA) and 1.625 μL of DNase/RNase-free water was added. The reaction was performed in an Eppendorf Mastercycler Gradient following such temperature program: 25°C for 5 min, 42°C for 60 min, and 70°C for 5 min. The cDNA was then diluted 1:4 with DNase/RNase-free water.

**Primer Design and Evaluation**

Primers were designed and as previously described [[2](#_ENREF_2)]. Briefly, primers were designed using Primer Express V3.0.2 with minimum amplicon size of 62 bp (amplicons of 100–120 bp were of superiority, if possible) and limited 3’ G + C percentage (Applied Biosystems). Primer sets were intentionally designed to fall across exon-exon junctions. Then, primers were aligned against NCBI database through BLASTN and UCSC’s COW (*Bos taurus*) Genome Browser Gateway to determine the compatibility of primers with already annotated sequence of the corresponding gene in both databases. Prior to qPCR, primers were verified through a 20-μL PCR reaction, which followed the same procedures of qPCR described below except the dissociation step. A universal reference cDNA amplified from all samples was utilized to ensure the identification of genes. Five microliters of PCR product was run in a 2% agarose gel stained with SYBER Safe (Cat. # S33102, Life Technologies, Grand Island, NY), and the remaining 15 μL were cleaned with a QIAquick PCR Purification Kit (Cat. #28104, Qiagen) and sequenced at the Core DNA Sequencing Facility of the Roy J. Carver Biotechnology Center at the University of Illinois, Urbana (in Supplemental Table 3). The sequencing product was confirmed through BLASTN at the National Center for Biotechnology Information (NCBI) database. Only primers that presented a single band of the expected size and the right amplification product were used for qPCR. The accuracy of a pair of primers was evaluated by the presence of a unique peak during the dissociation step at the end of qPCR. The details of primer sequences and the description of genes are shown in Supplemental Tables 1 and 2.

**Quantitative PCR (qPCR)**

qPCR was performed in a MicroAmp Optical 384-Well Reaction Plate (Cat. #4309849, Applied Biosystems, Grand Island, NY). Within each well, 4 μL of diluted cDNA combined with 6 μL of mixture composed of 5 μL 1×SYBR Green master mix (Cat. # 95073-05K, Quanta, Gaithersburg, MD), 0.4 μL each of 10 μ*M* forward and reverse primers, and 0.2 μL of DNase/RNase-free water were added. Three replicates and a 6-point standard curve plus the nontemplate control (NTC) were run for each sample to test the relative expression level. qPCR was conducted in ABI Prism 7900 HT SDS instrument (Applied Biosystems) following the conditions below: 10 min at 95°C, 40 cycles of 15 s at 95°C (denaturation), and 1 min at 60°C (annealing + extension). The presence of a single PCR product was verified by the dissociation protocol using incremental temperatures to 95°C for 15 s, then 65 °C for 15 s. The threshold cycle (Ct) data were analyzed and transformed using the standard curve with the 7900 HT Sequence Detection System Software (version 2.2.1, Applied Biosystems). Data were then normalized with the geometric mean of the three ICG as previously described by [[3](#_ENREF_3)].

**Relative mRNA Abundance of Genes within Adipose and Muscle Tissues**

Efficiency of qPCR amplification for each gene was calculated using the standard curve method (Efficiency = 10(–1/slope)). Relative mRNA abundance among measured genes was calculated as previously reported [[2](#_ENREF_2)], using the inverse of PCR efficiency raised to ΔCt (gene abundance = 1/EΔCt, where ΔCt = Ct of tested gene – geometric mean Ct of 3 internal control genes). Overall mRNA abundance for each gene among all samples of the same adipose tissue was calculated using the median ΔCt, and overall percentage of relative mRNA abundance was computed from the equation: 100 × mRNA abundance of each individual gene / sum of mRNA abundance of all the genes investigated (see Supplemental Table 4).

**Western Blot Analysis of PPARG within Adipose Tissue**

Western blotting was conducted to verify the key changes observed in protein expression of PPARG. The samples of SAT were homogenized in lysis buffer (#9803; Cell Signaling Technology, Inc., Danvers, MA) following the protocol of the company. The concentration of the total protein was measured using the BCA kit following the instruction of the company (#23225; Rockford, IL). Whole extracts (100 μg of total protein) were diluted with 5 × loading buffer (#161-0376, Bio-rad, Hercules, CA) and boiled at 95°C for 3 min. and were subject to 12% SDS/PAGE at 200 V until the dye front reached the bottom of the gel; in order to determine the size of the protein of interest the Precision Plus Protein WesternC Pack (#161-0385, Bio-rad, Hercules, CA) was loaded with the samples on every gel. The proteins were transferred to a PVDF membrane ((#IPVH00010, Millipore Corporation, Billerica, MA) using Trans-Blot SD Semi-Dry Electrophoretic Transfer Cell (#170-3940, Bio-rad). The PVDF membrane was blocked with 5% blotting-grade blocker (#170-6404, Bio-rad) dissolved in TTBS (0.05% Tween 20) for 60 min. at room temperature. The membrane was probed first with rabbit anti- PPARG antibody (Dilution, 1:400; #ab19481, Abcam, Cambridge, MA), rabbit anti-GAPDH antibody (Dilution, 1; 10000; #ab22555, Abcam). The PVDF membrane was incubated with the first antibody overnight at +4˚C on a VWR Rocking Platform Shakers (#40000-304, Radnor, PA). Following primary antibody incubation, membranes were washed with TBST for 3 times for 15 min. and then incubated in secondary antibody plus the StrepTactin-HRP conjugate detecting only Precision Plus Protein Western for 1 h at room temperature followed by the same wash step. The secondary antibody was goat polyclonal secondary antibody to rabbit IgG - H&L (HRP) (dilution 1: 10000; #ab6721, Abcam). Immunodetection using the Clarity Western ECL Substrate (#170-5060, Bio-rad) was performed according to the manufacturer’s instruction; the membrane was scanned using the ChemiDoc MP System (Bio-rad). The intensities of the bands were quantified [[4](#_ENREF_4), [5](#_ENREF_5)] using the ImageJ software and the expression of PPARG protein was normalized to the expression level of GAPDH protein.

**Table A.** GenBank accession number, sequence and amplicon size of primers used to analyze gene expression by quantitative PCR.

| **Accession no.** | **Gene** | **Primers**^1^ | **Primers (5’-3’)** ^2^ | **(bp)** ^3^ | **Source** |
| --- | --- | --- | --- | --- | --- |
| ***Glucose hemostasis and g*luconeogenesis** | | | | | |
| XM_590552.7 | *INSR* | F.3015 | CGGAGCTCAGAGATCACGACTAT | 106 | [[6](#_ENREF_6)] |
|  |  | R.3120 | AGGTTCACAGTTAAGTGCTCAGATGA |  |  |
| XM_003581871.1 | *IRS1* | F.4268 | TGTTGACTGAACTGCACGTTCT | 112 | [[7](#_ENREF_7)] |
|  |  | R.4379 | CATGTGGCCAGCTAAGTCCTT |  |  |
| NM_174604.1 | *SLC2A4* | F.261 | CCTTGGTCCTTGGCGTATTC | 102 | This manuscript |
|  |  | R.362 | TGTAGCTCTGTTCAATCACCTTCTG |  |  |
| NM_001113302.1 | *SREBF1* | F.1216 | TGTCCACAAAAGCAAATCGC | 101 | [[7](#_ENREF_7)] |
|  |  | R.1316 | TGTCGACCACCTCTGGCTTC |  |  |
| NM_174737.2 | *PCK1* | F.601 | AAGATTGGCATCGAGCTGACA | 120 | [[8](#_ENREF_8)] |
|  |  | R.720 | GTGGAGGCACTTGACGAACTC |  |  |
| XM_005226995.1 | *PC* | F.3497 | GCAAGGTCCACGTGACTAAGG | 124 | [[8](#_ENREF_8)] |
|  |  | R.3620 | GGCAGCACAGTGTCCTGAAG |  |  |
| NM_001101883.1 | *PDK4* | F.2621 | CAGGTGGACAGGGCAGTCTAG | 95 | [[9](#_ENREF_9)] |
|  |  | R.2715 | TCCTCCTTTTCCATCTTTCTTCTTT |  |  |
| ***Lipid metabolism nuclear receptors*** | | | | | |
| NM_181024.2 | *PPARG* | F.135 | CCAAATATCGGTGGGAGTCG | 101 | [[7](#_ENREF_7)] |
|  |  | R.235 | ACAGCGAAGGGCTCACTCTC |  |  |
| NM_001034036.1 | *PPARA* | F.729 | CATAACGCGATTCGTTTTGGA | 102 | [[10](#_ENREF_10)] |
|  |  | R.830 | CGCGGTTTCGGAATCTTCT |  |  |
| NM_001083636.1 | *PPARD* | F.1295 | TGTGGCAGCCTCAATATGGA | 100 | [[11](#_ENREF_11)] |
|  |  | R.1376 | GACGGAAGAAGCCCTTGCA |  |  |
| ***Lipid metabolism*** | | | | | |
| NM_173959.4 | *SCD* | F.809 | TCCTGTTGTTGTGCTTCATCC | 101 | [[2](#_ENREF_2)] |
|  |  | R.909 | GGCATAACGGAATAAGGTGGC |  |  |
| NM_174693.2 | *DGAT1* | F.190 | CCACTGGGACCTGAGGTGTC | 101 | [[2](#_ENREF_2)] |
|  |  | R.290 | GCATCACCACACACCAATTCA |  |  |
| NM_205793.2 | *DGAT2* | F.389 | CATGTACACATTCTGCACCGATT | 100 | [[2](#_ENREF_2)] |
|  |  | R.488 | TGACCTCCTGCCACCTTTCT |  |  |
| NM_001077909.1 | *INSIG1* | F.523 | CATCGACAGTCACCTTGGAGA | 108 | This manuscript |
|  |  | R.630 | TCCAGTTTAGCACTAGCGTGGT |  |  |
| NM_001012669.1 | *FASN* | F.6473 | ACCTCGTGAAGGCTGTGACTCA | 92 | [[2](#_ENREF_2)] |
|  |  | R.6564 | TGAGTCGAGGCCAAGGTCTGAA |  |  |
| XM_005227376.1 | *CPT1A* | F.1419 | TCGCGATGGACTTGCTGTATA | 100 | [[8](#_ENREF_8)] |
|  |  | R.1518 | CGGTCCAGTTTGCGTCTGTA |  |  |
| NM_001035289.3 | *ACOX1* | F.944 | CCATTGCCGTCCGATACAGT | 99 | [[11](#_ENREF_11)] |
|  |  | R.1042 | GTTTATATTGCTGGGTTTGATAATCCA |  |  |
| NM_174494.2 | *ACADVL* | F.707 | CCAGCCCCTGTGGAAAATACTA | 62 | [[12](#_ENREF_12)] |
|  |  | R.768 | GCCCCCGTTACTGATCCAA |  |  |
| ***Transcriptional transducers*** | | | | | |
| BC140488 | *ADIPOQ* | F.250 | GATCCAGGTCTTGTTGGTCCTAA | 131 | [[13](#_ENREF_13)] |
|  |  | R.380 | GAGCGGTATACATAGGCACTTTCTC |  |  |
| ***Post-transcriptional modifiers*** | | | | | |
| BC102884.1 | *SUMO1* | F.579 | TGGTGATCAAACCTCAGCCC | 104 | This manuscript |
|  |  | R.682 | CAAGCCTGCAAATGCACAGT |  |  |
| BC146107.1 | *UBC9* | F.494 | GGCCTACACGATCTACTGCC | 96 | This manuscript |
|  |  | R.589 | CCACAGGTCGCTGCTTATGA |  |  |
| ***Internal control genes*** | | | | | |
| NM_001025327.2 | *MTG1* | F.650 | CTTGGAATCCGAGGAGCCA | 101 | [[14](#_ENREF_14)] |
|  |  | R.550 | CCTGGGATCACCAGAGCTGT |  |  |
| BC108231 | *RPS15A* | F.31 | GAATGGTGCGCATGAATGTC | 101 | [[13](#_ENREF_13)] |
|  |  | R.131 | GACTTTGGAGCACGGCCTAA |  |  |
| NM_001034034.2 | *GAPDH* | F.275 | TGGAAAGGCCATCACCATCT | 53 | [[14](#_ENREF_14)] |
|  |  | R.327 | CCCACTTGATGTTGGCAG |  |  |
| NM_001101152.2 | *RPS9* | F.128 | CCTCGACCAAGAGCTGAAG | 64 | [[14](#_ENREF_14)] |
|  |  | R.191 | CCTCCAGACCTCACGTTTGTTC |  |  |
| NM_001037471.2 | *UXT* | F.300 | TGTGGCCCTTGGATATGGTT | 101 | [[14](#_ENREF_14)] |
|  |  | R.400 | GGTTGTCGCTGAGCTCTGTG |  |  |

^1^Primer direction (F – forward; R – reverse) and hybridization position on the sequence.

^2^ Primer sequence

^3^ Amplicon size in base pair (bp)

**Table B.** Gene symbol, gene name, and description of the main biological function and biological processes of the targets analyzed in subcutaneous adipose tissue and muscle.

| Symbol | Name | Cellular Localization | Summary description from NCBI |
| --- | --- | --- | --- |
| *ACADVL* | Acyl-CoA dehydrogenase, very long chain | Mitochondrial | Active toward esters of long-chain and very long chain fatty acids such as palmitoyl-CoA, mysritoyl-CoA and stearoyl-CoA. |
| *ACOX1* | Acyl-CoA oxidase 1, palmitoyl | Peroxisomal | Catalyzes the desaturation of very long chain acyl-CoAs to 2-trans-enoyl-CoAs. |
| *ADIPOQ* | Adiponectin | Extracellular space | Important adipokine involved in the control of fat metabolism and insulin sensitivity, with direct anti-diabetic, anti-atherogenic and anti-inflammatory activities. Stimulates AMPK phosphorylation and activation in the liver and the skeletal muscle, enhancing glucose utilization and fatty-acid combustion. |
| *CPT1A* | Carnitine palmitoyltransferase 1A | Mitochondrial | CPT1 is the key enzyme in the carnitine-dependent transport across the mitochondrial inner membrane and its deficiency results in a decreased rate of fatty acid beta-oxidation. |
| *DGAT1* | Diacylglycerol O-acyltransferase homolog 1 | ER membrane | Catalyzes the terminal and only committed step in triacylglycerol synthesis by using diacylglycerol and fatty acyl CoA as substrates. In contrast to DGAT2 it is not essential for survival. May be involved in VLDL (very low density lipoprotein) assembly. |
| *DGAT2* | Diacylglycerol O-acyltransferase homolog 2 | ER membrane | Essential acyltransferase that catalyzes the terminal and only committed step in triacylglycerol synthesis by using diacylglycerol and fatty acyl CoA as substrates. Required for synthesis and storage of intracellular triglycerides. |
| *FASN* | Fatty acid synthase | Cytosol | The enzyme encoded by this gene is a multifunctional protein. Its main function is to catalyze the synthesis of palmitate from acetyl-CoA and malonyl-CoA, in the presence of NADPH, into long-chain saturated fatty acids. |
| *INSIG1* | Insulin induced gene 1 | ER membrane | Oxysterols regulate cholesterol homeostasis through the liver X receptor (LXR)- and sterol regulatory element-binding protein (SREBP)-mediated signaling pathways. This gene is an insulin-induced gene. It encodes an endoplasmic reticulum (ER) membrane protein that plays a critical role in regulating cholesterol concentrations in cells. |
| *INSR* | Insulin receptor | Plasma membrane | Binding of insulin to the insulin receptor stimulates glucose uptake. |
| *IRS1* | Insulin receptor substrate 1 | Cytosol | This gene encodes a protein which is phosphorylated by insulin receptor tyrosine kinase. |
| *PC* | Pyruvate carboxylase | Mitochondrial | This gene encodes pyruvate carboxylase, which requires biotin and ATP to catalyse the carboxylation of pyruvate to oxaloacetate. The active enzyme is a homotetramer arranged in a tetrahedron which is located exclusively in the mitochondrial matrix. Pyruvate carboxylase is involved in gluconeogenesis, lipogenesis, insulin secretion and synthesis of the neurotransmitter glutamate. |
| *PCK1* | Phosphoenolpyruvate carboxykinase 1 | Cytosol and Mitochondrial | This gene is a main control point for the regulation of gluconeogenesis. The cytosolic enzyme encoded by this gene, along with GTP, catalyzes the formation of phosphoenolpyruvate from oxaloacetate, with the release of carbon dioxide and GDP. The expression of this gene can be regulated by insulin, glucocorticoids, glucagon, cAMP, and diet. |
| *PDK4* | Pyruvate dehydrogenase kinase, isozyme 4 | Mitochondrial | It is located in the matrix of the mitochondria and inhibits the pyruvate dehydrogenase complex by phosphorylating one of its subunits, thereby contributing to the regulation of glucose metabolism. Expression of this gene is regulated by glucocorticoids, retinoic acid and insulin. |
| *PPARA* | Peroxisome proliferator-activated receptor alpha | Nucleus | PPARalpha is a member of the nuclear receptor family of ligand-activated transcription factors that heterodimerize with the retinoic X receptor (RXR) to regulate gene expression |
| *PPARD* | Peroxisome proliferator-activated receptor delta | Nucleus | Receptor that binds peroxisome proliferators such as hypolipidemic drugs and fatty acids. Regulates the peroxisomal beta-oxidation pathway of fatty acids. Functions as transcription activator for the acyl-CoA oxidase gene |
| *PPARG* | Peroxisome proliferator-activated receptor gamma | Nucleus | Regulator of adipocyte differentiation |
| *SCD* | Stearoyl-CoA desaturase | ER membrane | Catalysis of the reaction: stearoyl-CoA + 2 ferrocytochrome b5 + O2 + 2 H+ = oleoyl-CoA + 2 ferricytochrome b5 + H2O involved in lipogenesis. |
| *SLC2A4* | Solute carrier family 4 (facilitated glucose transporter), member 4 | Plasma membrane, cytosol | This gene encodes a major glucose transporter. The encoded protein is found primarily in the cell membrane and on the cell surface. |
| *SREBF1* | Sterol regulatory element binding transcription factor 1 | Golgi and ER membranes, nucleus | It encodes a transcription factor that binds to the sterol regulatory element-1 (SRE1), which is a decamer flanking the low density lipoprotein receptor gene and some genes involved in sterol biosynthesis. |
| *SUMO1* | Small ubiquitin-like modifier 1 | Nucleus | It targets proteins as part of a post-translational modification system. It is involved in a variety of cellular processes, such as nuclear transport, transcriptional regulation, apoptosis, and protein stability. |
| *UBC9* | Ubiquitin-conjugating enzyme E2I | Nucleus | It targets the abnormal or short-lived proteins for degradation. This gene encodes a member of the E2 ubiquitin-conjugating enzyme family. |

**Table C.** Sequencing results obtained from PCR products.

| **Gene** | **Sequence** |
| --- | --- |
| ***ACADVL*** | GCAAGATTTGGATCAGTAACGGGGG |
| ***ACOX1*** | GGTNTTCNGTAACNACCCGGGGGAACCAGAACCACAGATTTTGGATTATCAAACCCAGCAATAGTAAACAAG |
| ***ADIPOQ*** | ACGCCCTTGCACTCCTTTGCAGAGAAAGGGATGCAGGTCTTCTTGGTCCTAAGGGTGAGACAGGAGATGTTGG |
| ***DGAT1*** | CGCAGCGATCCCTGTTCAGTTCTGACAGTGGCTTCAGCAACTACCGTGGCATCCTGAATTGGTGTGTGGTGATGCACA |
| ***DGAT2*** | GTCGTCGGTCCTTCTTCCCGTGCGTGGTTTGACTGGAACACACCCAAGAAAGGTGGCAGGAGGTCACAGTAATA |
| ***FASN*** | GCGAGACGTCTAGGTGTACGGGTGCCAGTCACGGATGCCCAGGATGTGAGTCACAGCCTTCAACGAGGTAAG |
| ***GAPDH*** | ATCTTCCAGGAGCGAGATCCTGCCAACATCAAGTGGG |
| ***INSIG1*** | CGCTGGCCCACTCGCGCTTGAACTTGTGTGGCTCTCCAAGGTGACTGTCG |
| ***INSR*** | CATGACTTCCTTCGTGACTTCAACGCAGGTGTTCCATTGGCCTATTTGACTTGTCATCTGAGCACTTAACTGTGAACCTTAAA |
| ***IRS1*** | ATCAGGCAGAAAAGCACTGTGACACCAGAACAATGAGTCTGCATAAACTTCATCTTCAACCTTAAGGACTTAGCTGGCCAACATGGAA |
| ***MTG1*** | CGAGCAGAACATCAGCGGCCGCTCACACACCTGAATCCTGGACATCACAGCTCTGGTGATCCCAGGA |
| ***PC*** | GATCATAGGAGTACAGAACTCATCTGGAAGAATCGAGTGACCACGCTGAGACTGGCAGCCTGACCATCCCCGACCCCTGCCTTCAGGACACTGTGCTGCCAGA |
| ***PCK1*** | GCCATGTGTACAGCAGTCGCATCATGACGAGGATGGGCACCAGCGTCCTGGAAGCGCTGGGGGACGGCGAGTTCGTCAAGTGCCTCCACAAA |
| ***PDK4*** | TTCTTCGTTTCGTCTCCGAGAGGAATGTGCTAGACTGCCCTGTCCACCTG |
| ***PPARA*** | AATTGAAGGCAGAAATCCTTACGTGTGAGCATGACCTAGAAGATTCCGAAACCGCGGA |
| ***PPARD*** | TTCTGTGCGGAGACCGGCCAGGCCTCAAGTATCGGTTCTCAGGTGGAGGCAACG |
| ***PPARG*** | TTTCTTTGAACGGAACTGGCCTGGGTGAAACTCTGGGAGATGCTCTTATTGACCCAGAGAGTGAGCCCTTCGCTGTCATTTTNNGGGGGCCGGCGACTCCCA |
| ***RPS15A*** | AAAAGAGAGGCAAACGCCAGGGTCCTTATTAGGCCGTG |
| ***RSP9*** | GCCAGTATGGGCTCCGGAACAAACGTGAGGTCTGGAGG |
| ***SCD*** | AGACTGTGCATGGTATCTGTGGGATGAAACGTTTCAAAACAGCCTGTTTTTTGCCACCTTATTCCGTATATAGCCAG |
| **SLC2A4** | TGCAGTTTGGCTACAACATTGGGGTCATCAATGCCCCCCAGAAGGTGATTGAACAGA |
| ***SREBF1*** | CGTCGTCCCCCACTGGTCTGGCTGATGAATGGGCTGCTGGTGCTCTTCTCCTTGGCACTTCTCTTTGTAAA |
| ***SUMO1*** | AATTACATGTGTGCATAGAGAGCCCGCCTTTTCCAGGACTGTGCATTTGCAGGCTTG |
| ***UBC9*** | AAGGGTTCGAGCACAAGCCAAGAAGTTTGCTCCCTCATAAGCAGCGACCTGTGG |
| ***UXT*** | CTGGTTATCGTGGAGCTCTCAGTTCATTGATCGTAAGAGCAGTCTCCTCACAGAGCTCAGCGACAATCTCAT |

**Table D.** qPCR performance among the genes measured in adipose tissue and muscle.

| **Gene** | **Median Ct^1^** | **Median ∆Ct^2^** | **Slope^3^** | **(R^2^)^4^** | **Efficiency^5^** |
| --- | --- | --- | --- | --- | --- |
| ***ACADVL*** | 26.01 | 4.23 | -3.28 | 0.99 | 2.02 |
| ***ACOX1*** | 23.48 | 2.28 | -3.06 | 0.99 | 2.12 |
| ***ADIPOQ*** | 20.28 | -2.20 | -3.34 | 0.99 | 1.99 |
| ***CPT1A*** | 27.00 | 4.36 | -3.16 | 0.99 | 2.07 |
| ***DGAT1*** | 27.41 | 4.80 | -3.30 | 1.00 | 2.01 |
| ***DGAT2*** | 23.48 | 2.28 | -3.06 | 0.99 | 2.12 |
| ***FASN*** | 26.02 | 3.27 | -3.38 | 0.99 | 1.98 |
| ***INSIG1*** | 19.54 | -2.38 | -3.11 | 0.99 | 2.10 |
| ***INSR*** | 23.79 | 2.49 | -2.88 | 0.99 | 2.22 |
| ***IRS1*** | 25.40 | 3.45 | -3.22 | 0.99 | 2.05 |
| ***PC*** | 26.42 | 3.68 | -3.54 | 0.99 | 1.91 |
| ***PCK1*** | 29.63 | 6.96 | -3.00 | 0.99 | 2.16 |
| ***PDK4*** | 26.42 | 3.68 | -3.54 | 0.99 | 1.92 |
| ***PPARA*** | 21.36 | -1.37 | -3.35 | 0.98 | 1.99 |
| ***PPARD*** | 24.49 | 2.79 | -3.69 | 0.99 | 1.87 |
| ***PPARG*** | 24.49 | 2.79 | -3.69 | 0.99 | 1.87 |
| ***SCD*** | 23.90 | 1.25 | -3.24 | 0.99 | 2.04 |
| ***SLC2A4*** | 27.22 | 5.65 | -3.03 | 0.98 | 2.14 |
| ***SREBF1*** | 20.52 | -1.12 | -3.18 | 0.99 | 2.06 |
| ***SUMO1*** | 24.50 | 3.58 | -2.97 | 1.00 | 2.17 |
| ***UBC9*** | 26.80 | 4.06 | -2.67 | 0.97 | 2.37 |

| ^1^ The median is calculated considering all time points and all cows. |
| --- |
| ^2^ The median of ∆Ct is calculated as [Ct gene – geometrical mean of Ct internal controls] for each time point and each steer. |
| ^3^ Slope of the standard curve. |
| ^4^ R^2^ stands for the coefficient of determination of the standard curve. |
| ^5^ Efficiency is calculated as [10^(-1 / Slope)^] |

**
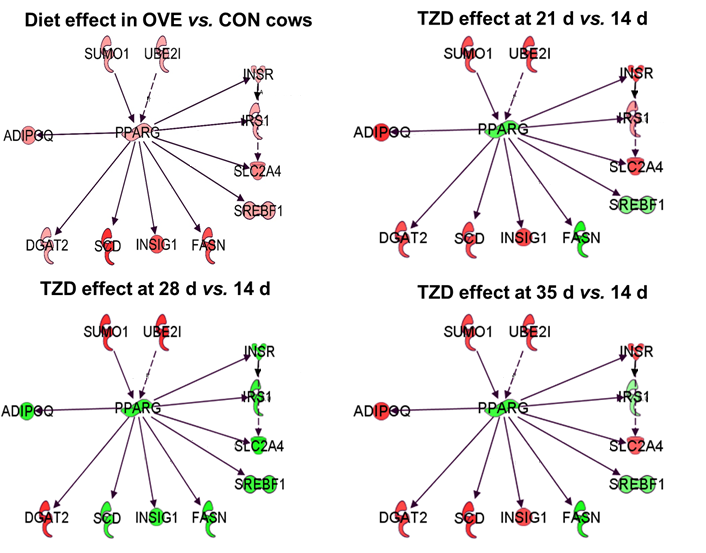
**

**Figure A.** Summary of potential regulatory network of genes involved in insulin response in subcutaneous adipose tissue for cows either fed moderate-energy diet (OVE, n=7) or a controlled-energy diet (CON, n=7) before TZD administration, during TZD administration and a washout period. Networks were developed using Ingenuity Pathway Analysis® (IPA) software (Ingenuity Systems, Redwood City, CA; [www.ingenuity.com](http://www.ingenuity.com)). Genes with a red- green-color were up-regulated and down-regulated by the specified treatment, respectively. Arrows in solid lines denote direct or arrows in dotted lines denote indirect interactions among genes. Fold change values for TZD effect were calculated considering the d 14 relative to diet initiation as control.

**
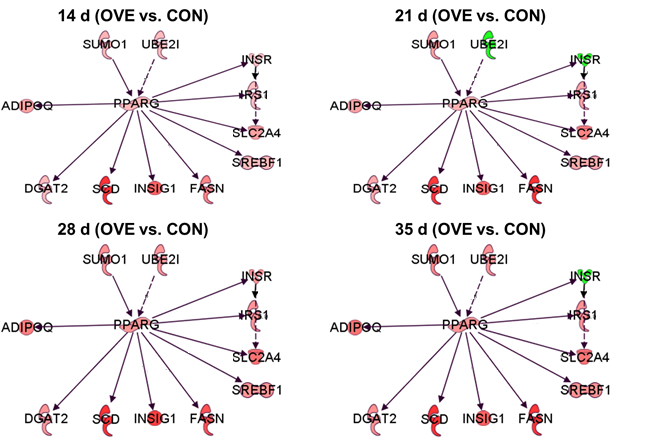
**

**Figure B.** Summary of Diet × Time effects on regulatory network of genes involved in insulin response in subcutaneous adipose tissue for cows either fed moderate-energy diet (OVE, n=7) or controlled-energy diet (CON, n=7) before TZD administration, during TZD administration and during a washout period. Networks were developed using Ingenuity Pathway Analysis® (IPA) software (Ingenuity Systems, Redwood City, CA; [www.ingenuity.com](http://www.ingenuity.com)). Genes with a red- green-color were up-regulated and down-regulated by the specified treatment, respectively. Arrows in solid lines denote direct or arrows in dotted lines denote indirect interactions among genes.

**
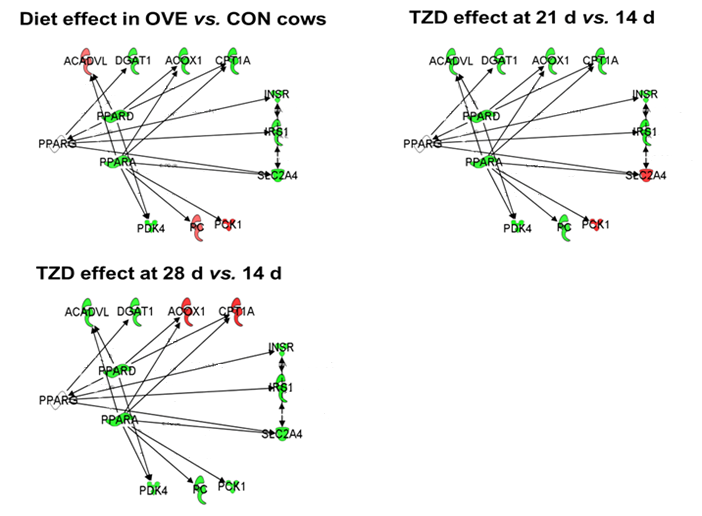
**

**Figure C.** Summary of potential regulatory network of genes involved in insulin response in muscle tissue for cows either fed moderate-energy diet (OVE, n=7) or a controlled-energy diet (CON, n=7) before TZD administration, and during TZD administration. Networks were developed using Ingenuity Pathway Analysis^®^ (IPA) software (Ingenuity Systems, Redwood City, CA; [www.ingenuity.com](http://www.ingenuity.com)). Genes with a red- green-color were up-regulated and down-regulated by the specified treatment, respectively. *PPARG* was not measured in muscle tissue. Arrows in solid lines denote direct or arrows in dotted lines denote indirect interactions among genes. Fold change values for TZD effect were calculated considering the d 14 relative to diet initiation as control.

**
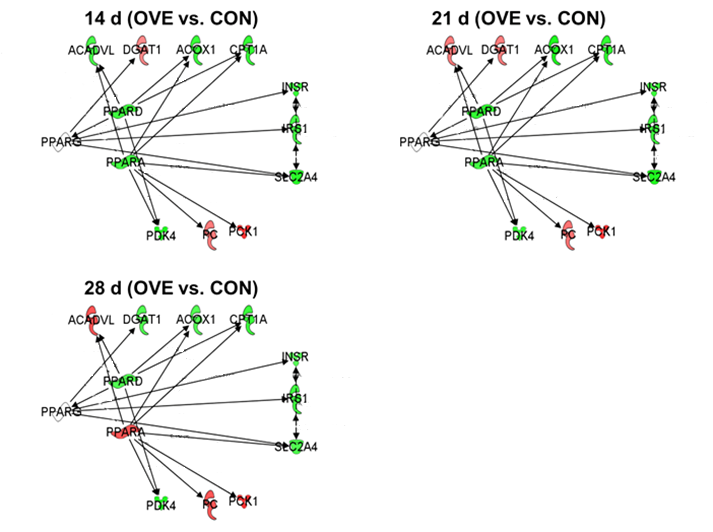
**

**Figure D.** Summary of Diet × Time effects on regulatory network of genes involved in insulin response in muscle tissue for cows either fed moderate-energy diet (OVE, n=7) or a controlled-energy diet (CON, n=7) before TZD administration and during TZD administration. Networks were developed using Ingenuity Pathway Analysis^®^ (IPA) software (Ingenuity Systems, Redwood City, CA; [www.ingenuity.com](http://www.ingenuity.com)). Genes with a red- green-color were up-regulated and down-regulated by the specified treatment, respectively. *PPARG* was not measures in muscle tissue. Arrows in solid lines denote direct or arrows in dotted lines denote indirect interactions among genes.

**References**

1. AOAC. AOAC. Official methods of analysis. Vol 1 and 2 Gaithersburg,. 2000;MD: AOAC International 2000.

2. Bionaz M, Loor JJ. Gene networks driving bovine milk fat synthesis during the lactation cycle. BMC genomics. 2008;9:366.

3. Vandesompele J, De Preter K, Pattyn F, Poppe B, Van Roy N, De Paepe A, et al. Accurate normalization of real-time quantitative RT-PCR data by geometric averaging of multiple internal control genes. *Genome Biol*. 2002;3:research0034-research.11

4. Gassmann M, Grenacher B, Rohde B, Vogel J. Quantifying Western blots: pitfalls of densitometry. Electrophoresis. 2009;30:1845-1855.

5. Tan HY, Ng TW. Accurate step wedge calibration for densitometry of electrophoresis gels. Optics Communications. 2008;281:3013-7.

6. Naeem A, Drackley JK, Stamey J, Loor JJ. Role of metabolic and cellular proliferation genes in ruminal development in response to enhanced plane of nutrition in neonatal Holstein calves. J Dairy Sci. 2012;95:1807-20.

7. Ji P, Osorio JS, Drackley JK, Loor JJ. Overfeeding a moderate energy diet prepartum does not impair bovine subcutaneous adipose tissue insulin signal transduction and induces marked changes in peripartal gene network expression. J Dairy Sci. 2012;95:4333-51.

8. Chen S. Effects of close-up dietary energy strategy and prepartal dietary monensin on hepatic mrna expression of enzymes involved in glucose and lipid metabolism in transition cows: University of Illinois; 2013.

9. Ji P. Transcriptional adaptation of adipose tissue in dairy cows in response to energy overfeeding: University of Illinois; 2011.

10. Graugnard DE. Immune function, gene expression, blood indices and performance in transition dairy cows affected by diet and inflammation: University of Illinois; 2011.

11. Loor JJ, Everts RE, Bionaz M, Dann HM, Morin DE, Oliveira R, et al. Nutrition-induced ketosis alters metabolic and signaling gene networks in liver of periparturient dairy cows. Physiol Genomics. 2007;32:105-16.

12. Thering BJ, Bionaz M, Loor JJ. Long-chain fatty acid effects on peroxisome proliferator-activated receptor-alpha-regulated genes in Madin-Darby bovine kidney cells: optimization of culture conditions using palmitate. J Dairy Sci. 2009;92:2027-37.

13. Piantoni P, Bionaz M, Graugnard DE, Daniels KM, Akers RM, Loor JJ. Gene expression ratio stability evaluation in prepubertal bovine mammary tissue from calves fed different milk replacers reveals novel internal controls for quantitative polymerase chain reaction. J Nutr. 2008;138:1158-64.

14. Bionaz M, Loor JJ. Identification of reference genes for quantitative real-time PCR in the bovine mammary gland during the lactation cycle. Physiol Genomics. 2007;29:312-9.
